# Towards Establishment of a Rice Stress Response Interactome

**DOI:** 10.1371/journal.pgen.1002020

**Published:** 2011-04-14

**Authors:** Young-Su Seo, Mawsheng Chern, Laura E. Bartley, Muho Han, Ki-Hong Jung, Insuk Lee, Harkamal Walia, Todd Richter, Xia Xu, Peijian Cao, Wei Bai, Rajeshwari Ramanan, Fawn Amonpant, Loganathan Arul, Patrick E. Canlas, Randy Ruan, Chang-Jin Park, Xuewei Chen, Sohyun Hwang, Jong-Seong Jeon, Pamela C. Ronald

**Affiliations:** 1 Department of Plant Pathology, University of California Davis, Davis, California, United States of America; 2 The Joint Bioenergy Institute, Emeryville, California, United States of America; 3 Plant Metabolism Research Center and Graduate School of Biotechnology, Kyung Hee University, Yongin, Korea; 4 Department of Plant Molecular Systems Biotechnology and Crop Biotech Institute, Kyung Hee University, Yongin, Korea; 5 Department of Biotechnology, College of Life Science and Biotechnology, Yonsei University, Seoul, Korea; 6 Plant Sciences, Centre for Cellular and Molecular Biology, Hyderabad, India; Iowa State University, United States of America

## Abstract

Rice (*Oryza sativa*) is a staple food for more than half the world and a model for studies of monocotyledonous species, which include cereal crops and candidate bioenergy grasses. A major limitation of crop production is imposed by a suite of abiotic and biotic stresses resulting in 30%–60% yield losses globally each year. To elucidate stress response signaling networks, we constructed an interactome of 100 proteins by yeast two-hybrid (Y2H) assays around key regulators of the rice biotic and abiotic stress responses. We validated the interactome using protein–protein interaction (PPI) assays, co-expression of transcripts, and phenotypic analyses. Using this interactome-guided prediction and phenotype validation, we identified ten novel regulators of stress tolerance, including two from protein classes not previously known to function in stress responses. Several lines of evidence support cross-talk between biotic and abiotic stress responses. The combination of focused interactome and systems analyses described here represents significant progress toward elucidating the molecular basis of traits of agronomic importance.

## Author Summary

A major limitation of crop production is imposed by a suite of abiotic and biotic stresses resulting in 30%–60% yield losses globally each year. In this paper, we used a yeast-based approach to identify rice proteins that govern the rice stress response. We validated the role of these new proteins using additional analyses to evaluate the function of these genes in rice and assessed whether they serve to positively or negatively regulate the stress response. This approach allowed us to identify ten genes that control resistance to bacterial disease and tolerance to submergence. The combination of approaches described here represents significant progress toward elucidating the molecular basis of traits of agronomic importance.

## Introduction

**Figure 1:**
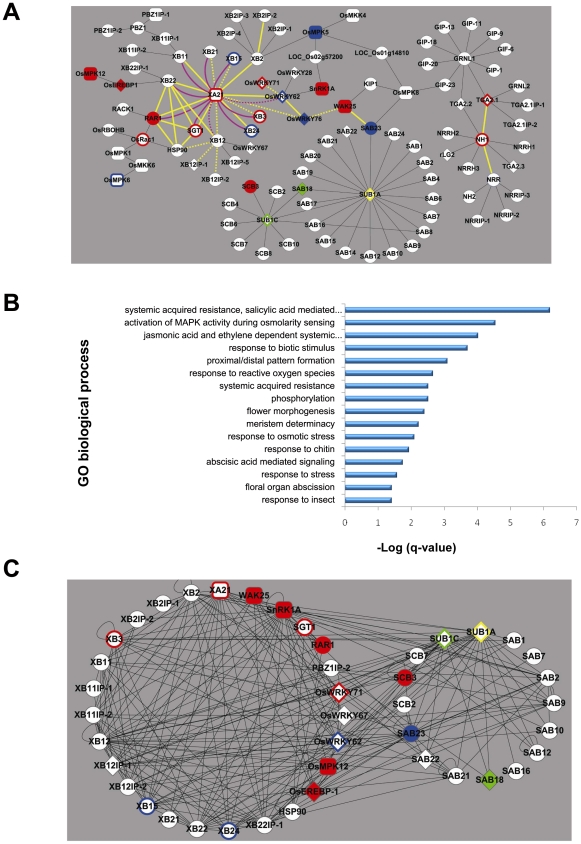
Construction, validation, and characterization of the rice stress-response interactome. (A) The XA21/NH1/SUB1 interactome as determined by Y2H cDNA library screening, interactions reported in the literature, and targeted Y2H assays (Text S1). Interactions shown by Y2H or in the literature, only, are represented by thin black edges (lines). Physical validation of the Y2H-based interactome was performed by either mating-based split ubiquitin system (purple edges: solid indicates an interaction was measured and dashed indicates no interaction was measured, Figure S1) or bimolecular fluorescence complementation (yellow edges: solid indicates an interaction was measured and dashed indicates no interaction was measured, Figure S2, Table S3). Response to *Xanthomonas oryzae* pv. *oryzae* (*Xoo*) challenge or submergence treatment was assessed for 24 members of the interactome (Text S1, Table S7). Nodes (proteins) that act as positive regulators of resistance to *Xoo* are shown in red (filled represent function shown in this study and outline represent function shown in the literature. Nodes that act as negative regulators of resistance to *Xoo* are shown in blue (filled: this study; outline: literature). Yellow and green nodes represent proteins that act as positive and negative regulators of tolerance to submergence, respectively (filled: this study; outline: literature). Nodes depicted as rounded rectangles and diamonds represent kinases and transcription factors, respectively. (B) Enrichment of gene ontology (GO) biological processes among interactome component proteins. The significance of enrichment for total of 1,042 GO terms was calculated by Fisher exact test, then obtained p-values were adjusted for multiple hypothesis testing by q-value [22]. Sixteen of 1,042 GO biological process terms were enriched by q <0.05 (represented as –log (q) in the bar graph, Table S2). (C) Protein-protein interaction map based on measurement of the matrix of interactions among and between 27 components of the biotic (XA21) stress-response and 16 components of the abiotic (SUB1) stress-response interactomes. Node colors and shapes are as in Figure 1A.

A major limitation of crop production is imposed by a suite of abiotic and biotic stresses resulting in 30%–60% yield losses globally each year [1]. The burgeoning field of systems biology provides new methodologies to make sense of plant stress responses, which are often controlled by highly complex signal transduction pathways that may involve tens or even thousands of proteins [2]. Complementary to large-scale approaches to delineate organisms' entire interactomes [3], we have developed a focused, high-quality Y2H-based interactome around the following key proteins that control the rice responses to disease and flooding: XA21 [4], NH1 (*NPR1 homolog1/OsNPR1*) [5], [6], SUB1A and SUB1C (submergence tolerance 1A, 1C) [7] (Figure 1A, Table S1). XA21 is a host sensor (also called a pattern recognition receptor (PRR)) of conserved microbial signatures that confers resistance to the Gram-negative bacterium *Xanthomonas oryzae* pv. *oryzae* (*Xoo)* [4], [8], [9]. Overexpression of *Nh1* in rice also enhances resistance to *Xoo* [5]; whereas reduced expression of *Nh1* impairs benzothiadiazole-induced resistance to *Pyricularia oryzae* [10]. SUB1A and SUB1C are ethylene response transcription factors that regulate response to prolonged foliar submergence [7]. Much remains to be learned about the signaling pathways controlled by these pivotal stress response proteins.

To identify components of these signaling pathways, we carried out yeast two hybrid screening to construct a rice response interactome. We then validated the robustness of the interactome using bimolecular fluorescence complementation [11], yeast mating-based split ubiquitin system assays [12], and phenotypic analysis. Transgenic analysis of genes encoding key proteins coupled with correlation analysis of transcriptomics data and protein-protein interactions revealed ten interactome members that function as positive or negative regulators of biotic or abiotic stress tolerance in rice. Fourteen additional members of the interactome have previously been reported to function in stress tolerance. The high-quality interactome and systems-level analyses described here represent significant progress toward elucidating the molecular basis of traits of agronomic importance.

## Results/Discussion

### Construction of the rice stress-response interactome

We initially reconstructed four separate sub-interactomes for NH1, the intracellular kinase domain of XA21 (termed XA21K668 [13]), SUB1A, and SUB1C by screening a rice cDNA library pool. Subsequent rounds of screening with identified interactors, targeted assays with additional proteins identified based on sequence homology, and inclusion of connections from the rice kinase interactome [14] revealed that the NH1-, XA21-, and SUB1-anchored interactomes form a single rice stress interactome (Figure 1A, Table S1).

The four sub-interactomes were constructed by using a high throughput yeast two hybrid (Y2H) approach to identify components of the XA21-, NH1-, and SUB1- signaling pathways. We identified a total of 8 unique XA21 binding proteins (XBs, Table S1). Five of these XBs, XB2, XB10 (hence forth called OsWRKY62), XB11, XB12 and XB22, were chosen for further screening as baits in the Y2H to identify XB interacting proteins (XBIPs). Using Arabidopsis NPR1 as bait, six interacting proteins (NRR, NRRH1, rTGA2.1, rTGA2.2, rTGA2.3, and rLG2) were isolated by the same approach as described above. With NRR as bait, we isolated an additional six proteins (NH1, NH2, NRRIP-1, NRRIP-2, and NRRIP-3). With rTGA2.1 as bait, 4 interacting proteins were identified (TGA2.1IP-1, TGA2.1IP-2, GRNL1 and GRNL2). GRNL1 was used as bait to isolate nine interacting proteins (rTGA2.1, rTGA2.2, GIP-1, GIP-6, GIP-9, GIP-11, GIP-13, GIP-18, GIP-20, and GIP-23). Using SUB1A and SUB1C as baits, we identified 20 SUB1A binding proteins (SABs) and 9 SUB1C binding proteins (SCBs) (Table S1). Two proteins, SAB8 (SCB5) and SAB18 (SCB9), were identified using both SUB1A and SUB1C as baits. All identified proteins were repeatedly confirmed through secondary screenings were further characterized.

Additional proteins were incorporated into the XA21 and NH1/NRR interaction based on literature curation and subsequent experimentation. For example, ten interactors identified through our previous rice kinase Y2H screen [14], were incorporated into the the rice stress response interactome (Figure 1A, Table S1). We also demonstrated, through Y2H and co-immunoprecipitation assays, that OsRac1 (rice small GTPase, previously shown to play an important role in the rice defense response) interacts with RAR1 (required for *Mla1*2 resistance), HSP90 (heat shock protein 90), OsRBOHB (rice respiratory burst oxidase homologB), and OsMPK1 [15], [16], [17]. We also showed that OsMPK12 (blast- and wound-induced MAP kinase (BWMK1)), which was previously demonstrated to be induced upon infection by *Magnaporthe grisea*), interacts with XB22IP-2 (hereafter, called OsEREBP1 (rice ethylene-responsive element-binding protein 1, AP2)) [18]. We tested additional interactions based on of the presence of predicted protein motifs. For example, a tetratricopeptide repeat domain found in XB22 is also found in SGT1 (Suppressor of G-two allele of *Skp1*). XB12 shows sequence similarity with p23, a protein that modulates Hsp90-mediated folding of key molecules involved in diverse signal transduction pathways [19]. We therefore tested the protein interactions of these two XBs with components of the HSP90/SGT1/RAR1 chaperone complex [20]. Positive interactions were incorporated into the rice stress response interactome. Similarly, because NH1 interacts with NRR, we tested two predicted paralogs (NRRH1 and NRRH2) with NH1.

While a genetic interaction between the NH1 and XA21 signaling pathways has previously been demonstrated [21], signaling components shared between submergence tolerance and *Xoo*-resistance have not yet been described. The current network is composed of 100 proteins and shows significant enrichment (by *q*<0.05, Fisher exact test with multiple hypothesis adjustment [22]) for several gene ontology (GO) terms related to both abiotic and biotic stress responses (Figure 1B, Table S2). Among molecular functions, the rice stress response interactome is particularly rich in transcription factors (diamond nodes in Figure 1A, *p*-value  = 7.1×10^−5^, Fisher exact test), including 5 WRKY proteins, 4 TGA proteins, and 4 AP2 factors.

### Validation of the interactome using *in vivo* assays

Validation of subsets of protein-protein interactions (PPIs) with two additional *in vivo* assays provides evidence that the interactome is of high quality. Using a mating-based split ubiquitin system that measures interactions with transmembrane proteins [12], we confirmed that 80% (8 out of 10 tested) of the XA21-binding (XB) proteins are able to interact with the full-length, membrane-spanning XA21 (the initial screen was conducted with the truncated XA21K668 protein) (Figure 1A, Figure S1). To assess whether the observed Y2H protein-protein interactions occur in plant cells, we examined 30 candidate proteins pairs using bimolecular fluorescence complementation (BiFC) in rice protoplasts. To rule out false-positive interactions, we tested the interaction of each protein with negative control vectors consisting of half of the yellow fluorescent protein. We found that 14 of the 30 tested showed interactions as detected by fluorescence only in the presence of the interacting rice protein but not in the presence of the negative control. Four proteins fluoresced in the presence of the negative control but displayed greatly enhanced fluorescence intensity in the presence of the interacting rice protein indicating that the interaction could be reproduced *in vivo*. Together these results indicate that 60% (18/30) of the tested pairs of interactome members interact in rice protoplasts as revealed by BiFC assays (Figure 1A, Figure S2, Table S3).

### Interactions among interactome components

Components showing a large number of interactions with other interactome members (high degree) have been hypothesized to be essential for survival of the organism [23] although this finding has been disputed [24]. To identify such key hub proteins, we identified components in the rice stress interactome that displayed high degrees of interactions and then subjected them to pair-wise PPI assays. We tested a 24×20 matrix of 27 biotic stress (XA21) interactome components, a 14×14 matrix of 16 abiotic stress (SUB1) interactome components, and a 24×16 matrix of biotic-abiotic interactome components (Text S1, Table S4). An interaction was considered significant and reproducible if we observed it was replicated in two to three independent assays (Table S4).

Pair-wise PPI assays among interactome members revealed large numbers of possible interactions within and between the biotic and abiotic sub-interactomes (average degree 11±8, Figure 1C, Table S4). These interactomes have a high percentage (21.8%) of interactions beween their components (232 interactions out of 1060 tested) (Table S4). The biotic stress response interactome exhibits the highest level of interactions at 27.5% (132/480). The abiotic stress response interactome and the union between the biotic-abiotic stress response interactomes are even more highly connected [18.9% (37/196) and 16.4% (63/384), respectively]. The high number of interactions observed in the stress response interactome suggests that a large fraction of the components are capable of interacting with each other. These results also suggest that these components serve as members of large and/or changing complexes *in vivo* [25].

While the high number of interactions we observed is an order of magnitude greater than observed for studies of large-scale interactomes [3], it is comparable to smaller scale, more focused studies, such as that carried out for Arabidopsis MADS box transcription factors. In the MADS-box factor study, an average of only 5.4% of the components showed interactions (272/4998). However, when transcription factors predicted to function in the same biological process were examined, they displayed an increased number of interactions. For example, MADS-box factors predicted to be involved in floral development showed >15% interactions [26].

Consistent with their demonstrated key roles in response to stress, XA21, SUB1A, and SUB1C exhibit a high degree of interactions. In the matrix-based PPI tests, each of these interacted with over 10 additional proteins not initially identified as interactors in the original screen (Table S4). Other proteins with published roles in biotic stress signaling, including XB15 [13], XB3 [27], OsWRKY62 [28], and XB24 [29] are also among those with an above average degree of interaction. Such hubs may have a higher chance of engaging in essential functions because they participate in more interactions [30].

### Expression analysis of interactome components

**Figure 2:**
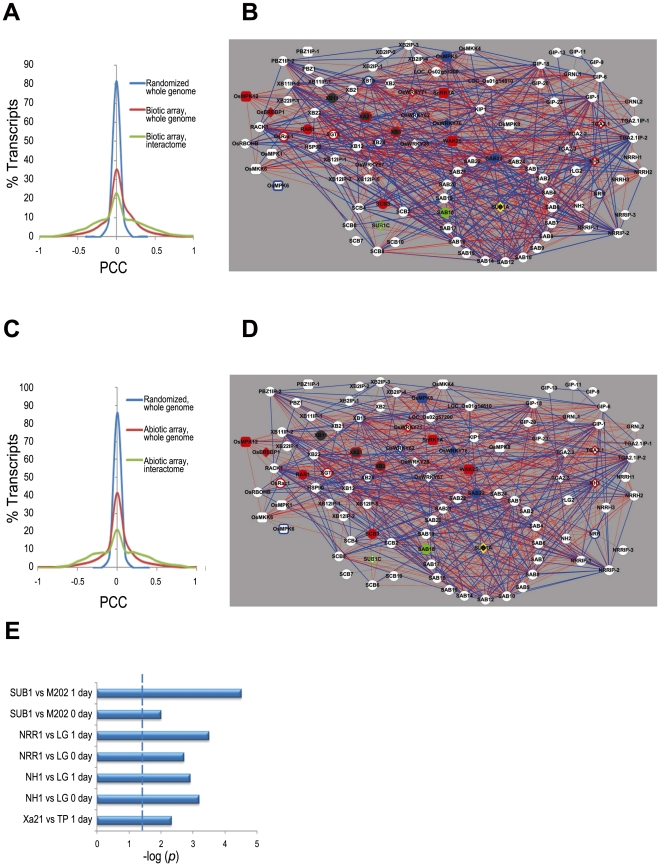
Transcriptome context for the rice stress interactome. (A) Distribution of Pearson's correlation coefficient (PCC) values calculated from the 179 biotic stress Affymetrix arrays data (listed in Table S5) for the interactome components only (green line), all genes in the rice genome (red line) and all rice genes with the array data randomized (blue line), demonstrate that the expression of the interactome members is highly correlated compared to that of all rice genes. (B) Coexpression network of interactome based on the biotic stress arrays (listed in Table S5). Red edges indicate positive correlations (PCC > 0.5) and blue edges indicate negative correlations (PCC <−0.5). Node shapes and colors are as in Figure 1A except the purple filled nodes, which indicates the genes for which we were unable to calculate PCC due to lack of unique probes. (C) Distribution of PCC as for (A) but with the abiotic stress Affymetrix arrays (Table S5) (D) Coexpression network as for (B) but with the abiotic stress arrays. (E) Enrichment test of interactome genes in NSF45K array data by Fisher exact test. The significance level of p-values <0.05 is indicated by dashed line. M202 vs. *Sub1*A::*Sub1A* vs. is a comparison of the cultivar M202 with a near isogenic line in which the *Sub1* locus has been introgressed [32]. LG vs. *Ubi*::*Nrr* is a comparison of the cultivar LiaoGeng (LG) and LG transgenic line #64 that overexpresses NRR from the maize ubiquitin promoter. LG vs. *Ubi*::*Nh1* is a comparison of LG and LG transgenic line #11that overexpresses NH1. TP vs. *Xa21::Xa21* is a comparison of the cultivar Taipei309 (TP) and TP transgenic line #106-17-3-37 that expresses Xa21 from the Xa21 native promoter. ‘0 day’ indicates that the sample was taken immediately before stress initiation (i.e., submergence or *Xoo*-inoculation). ‘1 day’ indicates that the sample was taken approximately 24 hours after application of stress.

Coexpression network analysis and stress-specific transcriptomics of the interactome components support the validity of the interactome as an integrated module and highlights specific nodes that may function in cross-talk between the abiotic and biotic stress responses (Figure 2). The interactome is highly enriched for genes with correlated or anticorrelated expression compared with the whole genome (Figure 2A and 2C). For this analysis, we built rice biotic and abiotic stress gene transcript coexpression networks for the interactome members based on Pearson's correlation coefficients (PCC) calculated from publically available Affymetrix microarray data (Table S5). We define a correlated or anticorrelated interaction by PCC > |0.5|, a criterion under which 15% of interactome gene pairs interact, compared with ∼5.5% of pairs in the whole rice genome, and no pairs when the expression profiles are randomized (Figure 2A and 2C, Table S5). In both the coexpression networks derived from the abiotic and biotic microarray datasets, many components of the SUB1A (abiotic stress) and the XA21/NH1 (biotic stress) sub-interactomes display highly correlated or anticorrelated expression (Figure 2B and 2D, Table S5). This result further supports cross talk between the abiotic and biotic response networks. Contrasting the networks built from the different array sets, reveals that only a fraction of edges are conserved between the biotic and abiotic gene expression networks. This suggests that the expression of interactome members, and thus their availability to form PPIs with each other, varies depending on the stress regime, consistent with a model of dynamic complex formation [31] (Figure 2B and 2D).

**Figure 3:**
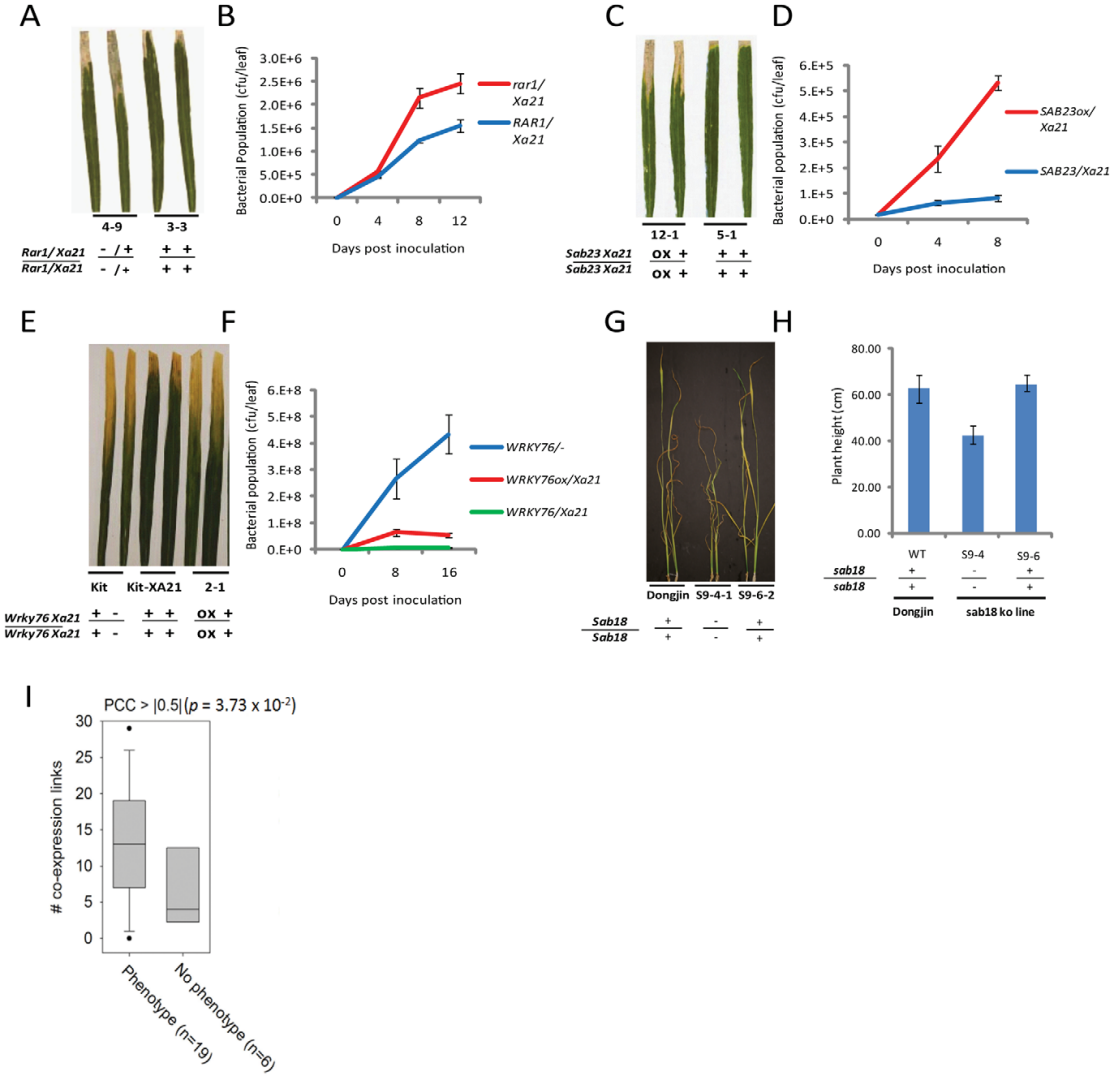
Representative evidence that interactome components function in rice stress responses. (A–B) Challenge of *rar1* (knockout)/*Xa21* (IRBB21) F_2_ segregants with *Xoo* (PR6) reveals that RAR1 is a positive regulator of XA21 signaling (see also Figure S6). (A) Water-soaked disease lesions 14 days post inoculation (dpi) of *rar1*/Xa21 leaves (plant 4–9) compared to *Rar1*/*Xa21* leaves (plant 3-3). (B) *Xoo* population growth over 12 days of infection from three representative leaves per time point from rar1/Xa21 vs. Rar1/Xa21 F_3_ segregants. (C–D) Challenge of Ubi::Sab23/Xa21 (IRBB21) F_3_ segregants with *Xoo* reveals that SAB23 negatively regulates XA21-mediated defense (see also Figure S7). (C) Water-soaked disease lesions 14 dpi of Ubi::Sab23/Xa21 leaves (plant 12-1) compared with Xa21 leaves (plant 5-1). (D) Xoo population growth over 12 days of infection from three representative leaves per time point from Ubi::Sab23/Xa21 vs. Xa21 F_3_ segregants. (E–F) Challenge of T_2_ Ubi::Wrky76/Xa21 Kitaake (Kit) plants with Xoo reveals that WRKY76 negatively regulates XA21-mediated defense (see also Figure S11). (E) Water-soaked disease lesions 14 dpi of Ubi::Wrky76/Xa21 leaves (plant 2-1) compared to Xa21-Kit leaves. (F) Xoo population growth over 14 days of infection from three representative leaves per time point from Ubi::Wrky76/Xa21-Kit T_1_ plants vs. Xa21-Kit. (G–H) Submersion of sab18 (knockout) plants reveals that SAB18 functions as a negative regulator of submergence tolerance (see also Figure S13). (G) Shoot elongation response of sab18 Dongjin (plant S9-4-1) compared to Dongjin (wild type) and null segregant (S9-6-2) after 14 days of submergence (H) Shoot elongation of sab18 Dongjin (line S9-4) compared with sab 18 null segregant (S9-6) and wild type after 14 days of submergence. (I) Degree distributions by coexpression network, in which links are defined by PCC > |0.5| based on 219 abiotic microarrays, for interactome genes with phenotypic effect or no phenotypic effect. Genes encoding interactome components with phenotypic effects show a significantly higher degree distribution than genes with no phenotypic effect (p<0.04, Wilxoson signed rank test).

We also generated microarray data to monitor transcriptional responses of *Xa2*1-expressing and *Nh1*- and *Nrr*-overexpressing rice (NRR binds NH1 and is a negative regulator of resistance [21]) before and after *Xoo* infection. Analysis of this dataset as well as a previously reported *Sub1a*-specific response dataset [32], reveals that interactome members are significantly enriched among differentially expressed genes (*p*<0.05, Fisher exact test, Figure 2E, Figure 3, Table S6, Figure S3).

### Phenotypic assays of key interactome components

The interactome includes fourteen components that have previously been shown to regulate resistance to *Xoo,* further supporting the high quality of the interactome (Figure 1A, Table S7). We measured the *Xoo* and/or submergence response phenotypes of mutant rice lines for twenty additional interactome members, focusing primarily on genes encoding proteins with a high degree of PPIs (Table S7). Note that because of this bias in our experimental design, we are unable to test for correlation between a high degree of PPIs and a functional role in rice stress tolerance. Our phenotypic results show that nine out of seventeen genes (53%) that we assayed for a role in resistance to *Xoo* showed altered defense response phenotypes. Only one out of nine genotypes assayed showed altered tolerance to submergence, possibly due to the absence of SUB1A in the genotypes we examined (Table 1, Figure 3A–3H, Figures S4, S5, S6, S7, S8, S9, S10, S11, S12, S13).

**Table 1:** Summary of the 10 interactome components that display altered phenotypes in response to *Xanthomonas oryzae* pv. *oryzae* (*Xoo*) or submergence treatment.

Name	Locus IDPutative Function*	Genotype	Phenotype	Regulatory class
RAR1	LOC_Os02g33180CHORD family disease-resistance protein	5 segregating F_3_ families of Dongjin-RAR1 knockout X IRBB21 (XA21)	Enhanced susceptibility to *Xoo*	(+) disease resistance, XA21-dependent
OsEREBP-1	LOC_Os02g54160AP2 transcription factor	Overexpression of OsEREBP-1 in Kitakke	Enhanced resistance to *Xoo*	(+) disease resistance
WAK25	LOC_Os03g12470Wall-associated receptor kinase	Overexpression and RNAi of WAK25 in Kit-XA21	OX: Enhanced resistance to *Xoo;*RNAi: Enhanced susceptibility to *Xoo*	(+) disease resistance, XA21-dependent
SCB3	LOC_Os03g14120Dihydrodipicolinate reductase	Overexpression of SCB3 in LiaoGeng	Enhanced resistance to *Xoo*	(+) disease resistance
SnRK1A	LOC_Os05g45420Sucrose non-fermenting-1-related protein kinase-1	RNAi of SnRK1A in Kit-XA21	Enhanced susceptibility to *Xoo*	(+) disease resistance, XA21 dependent
OsMPK12	LOC_Os06g49430Mitogen-activated protein kinase	Knockout of OsMPK12 in Dongjin	Enhanced susceptibility to *Xoo*	(+) disease resistance
OsMPK5	LOC_Os03g17700Mitogen-activated protein kinase	RNAi of OsMPK5 in Nipponbare	Enhanced resistance to *Xoo*	(−) disease resistance
OsWRKY76	LOC_Os09g25060WRKY transcription factor	Overexpression of OsWRKY76 in Kit-XA21	Enhanced susceptibility to *Xoo*	(−) disease resistance, XA21-dependent
SAB23	LOC_Os12g32980PHD domain protein	3 segregating F_3_ families of Dongjin-SAB23 Activation X IRBB21 (XA21)	Enhanced susceptibility to *Xoo*	(−) disease resistance, XA21-dependent
SAB18	LOC_Os11g06410SANT domain transcription factor	Knockout of SAB18 in Dongjin	Enhanced tolerance to submergence	(−) submergence tolerance

*Putative function determined by BLASTP search.

Importantly, our phenotypic analysis revealed roles for two protein classes that, to our knowledge, were previously unknown to function in the plant stress response. on sequence similarities, SAB18 is a SANT-domain transcription factor, and, SCB3, is an enzyme involved in lysine biosynthesis (Table 1). SAB18 is a negative regulator of submergence tolerance suggesting that it may modulate the antagonistic activities of its two binding partners, SUB1A and SUB1C (Figure 3G and 3H, Figure S13). SCB3 serves as a positive regulator of resistance to *Xoo* (Figure S8). This result together with an earlier report showing that lysine levels increase in the *Xoo*-challenged Xa21 rice compared to mock treated controls [33], suggests that lysine plays an important, although undefined, role in the rice innate immune response.

The remaining eight proteins that we demonstrate to be involved in rice innate immunity have similarity to known stress-response factors (Table 1, Table S7, Text S1). Though many of these proteins were identified due to association with XA21 or an XB, modification of the expression of four of these genes gives altered resistance phenotypes in the absence of XA21 (Table 1), suggesting that they function in multiple biotic stress-response signaling pathways. Of particular significance, knockdown or knockout experiments show a role for three proteins, (RAR1, WAK 25 (wall associated kinase 25), and SnRK1 (sucrose non-fermenting-related protein kinase 1)), in XA21-mediated immunity.

The chaperone complex, HSP90/RAR1/SGT1 has been long known to play a positive role in intracellular NBS-LRR-mediated immunity [34]. RAR1 and HSP90 have also been shown to play a role in Arabidopsis FLS2-mediated signaling [35] and maturation of the rice chitin extracellular receptor OsCERK1 [36], respectively. Our observation that RAR1 serves as a positive regulator of XA21-mediated immunity (Figure 3A and 3B, Figure S6) further affirms that this complex contributes to host sensor-mediated immunity.

*Wak25* (LOC_Os03g12470), compromises XA21-mediated immunity (Figure S10), indicating that WAK25 is a positive regulator of this process. WAKs have previously been shown to function as positive regulators of plant defense responses [37]. Although we do not yet know how WAK25 serves to regulate XA21-mediated immunity, there is precedence for interaction of PRRs with other receptor kinases. For example, the Arabidopsis FLS2 PRR interacts with the BRI1-associated kinase (BAK1) to transduce the immune response [38].

We also found that OsMPK5, previously demonstrated to serve as a negative regulator of resistance to the fungus, *Magnaporthe grisea*, and the bacteria, *Burkholderia glumae* [39], also negatively regulates resistance to *Xoo* (Figure S4). In contrast, the *Arabidopsis* protein with highest similarity to OsMPK5, AtMPK3, acts downstream of the *Arabidopsis* host sensor FLS2 and is a positive regulator of camalexin-mediated resistance to *Botrytis cinera* [40], [41]. The opposite regulatory roles for these *Arabidopsis* and rice predicted MPK orthologs underlines the limitations of extrapolating function between plant species.

OsMPK12 -and OsEREBP1 - are also positive regulators of resistance to *Xoo* (Figure S5, Figure S12). OsMPK12 was previously shown to phosphorylate OsEREBP1 [18]. OsEREBP1, as phosphorylated by OsMPK12, exhibits enhanced binding to the GCC box element of pathogenicity-related (PR) gene promoters. Overexpression of OsMPK12 in tobacco enhances expression of PR genes and increases resistance to *Pseudomonas syringae* and *Phytophthora parasitica* infection [18]. Thus, our results together with previously published studies indicate that OsMPK12 and OsEREBP1 are positive regulators of resistance to many pathogens.

We have also demonstrated a negative regulatory function for OsWRKY76 (Figure 3E and 3F, Figure S11), as has previously been shown for OsWRKY62 [28]. These two OsWRKYs are in the same WRKY subgroup (IIA) and are orthologs of barley HvWRKY1 and HvWRKY2, which serve as negative regulators of resistance to *Blumeria graminis* [42]. Along with our observation that the OsWRKY IIA proteins interact with members of the XA21 and SUB1 sub-interactomes [28], [43], these data are consistent with the WRKYIIA proteins playing a key role in fine-tuning grass defense responses.

SAB23 is a plant homeobox domain- (PHD) containing protein, which is known to function in development [44] and has been linked to response to pathogen stress [45] (Table 1). SAB23 serves as a negative regulator of resistance to *Xoo* (Figure 3C and 3D, Figure S7). This result supports previous observations that components regulating XA21-mediated resistance are also involved in developmental regulation [21], [46], [47]

SnRK1A, a well-known regulator of sugar sensing [48], was identified as a positive regulator in XA21-mediated immunity (Figure S9). *Arabidopsis* SnRK1 has been identified as a key regulator in sugar sensing and abscisic acid (ABA) signaling [49]. Though ABA has typically been found to act as a positive regulator of abiotic stress responses and a negative regulator of biotic stress responses [50], several positive regulators of the rice biotic stress response including SnRK1A and OsMPK12 participate in ABA signaling. Genes with ABA-related GO annotations are also up-regulated in *Nh1*-overexpressing and *Sub1a*-expressing transgenic rice (*q* = 1.3×10^−2^ and *q* = 5.3×10^−10^, respectively, Fisher exact test, multiple hypothesis adjustment) (Table S9). Together these observations support the hypothesis that ABA also has important functions in resistance to *Xoo* and tolerance to submergence in rice.

Comparable to analyses that show a correlation between essentiality and network degree centrality for essential genes [51] and negative regulators of growth (i.e., tumor suppressors) [52], we found that the rice interactome proteins with a validated role in the stress response have a significantly higher degree centrality in the abiotic co-expression network compared with those for which we were unable to measure a phenotype (Figure 3i, *p* = 3.7×10^−2^, Wilcoxon signed rank test, Table S8). Thus, interactome members that serve as central hubs as measured by co-expression analysis are more likely to function in the stress response than those members that do not serve as central hubs. This observation indicates the power of using the “guilt-by-association principle” to guide experiments based on co-expression maps [53], [54].

### Conclusions

Here, we constructed a rice stress response interactome composed of 100 proteins governing the rice response to biotic and abiotic stress. Integration of protein-protein interaction assays, co-expression studies, and phenotypic analyses allowed us to efficiently identify ten novel proteins regulating the rice stress response.

## Materials and Methods

### Yeast two-hybrid screening

The XA21 kinase fragment K668 was cloned into the Y2H bait vector pMC86. SUB1A and SUB1C were also cloned into pMC86. Sequence information is provided in Table S1. The Y2H screening experiments for SUB1A and SUB1C were conducted in the same manner as those for XA21. Bait constructs were transformed into yeast strains HF7c MATa, plated on selective medium, and screened as described (Clontech's Matchmaker Pretransformed Libraries User Manual). Colonies from the HF7c baits were grown to approximately 2×10^8^ cfu/mL in 50 mL synthetic dextrose (SD: 6.7 g Difco yeast nitrogen base w/o amino acids, 2% glucose, 1X drop out solution [supplemented with appropriate amino acids], pH 5.8) lacking Tryptophan (Trp) media for use in the primary screens. Cells of HF7c baits were pelleted, washed once with sterile H_2_O and resuspended in 50 mL rich yeast media, YPAD (20 g Difco peptone, 10 g yeast extract, 40 mg Adenine hemisulfate, 2% glucose, pH 5.8). Target yeast (Y187) were transformed with cDNAs from a Hybrizap (Stratagene) Y2H library derived from seven-week-old IRBB21 (Indica cultivar containing *Xa21*) leaf mRNA. One aliquot of the Y187 target yeast was mixed with the Hf7c bait yeast in 50 mL YPAD and poured into a tissue culture flask. Yeast strains were allowed to mate for 20 to 24 hrs at 28°C with slight shaking. Yeast were then isolated and washed twice with sterile water and plated on SD medium lacking Histidine (His), Tryptophan (Trp), Leucine (Leu) and supplemented with 2 mM 3-amino-1, 2, 4-triazole (3-AT). Putative positive diploids from the primary screens were isolated and plasmids extracted. Confirmation of interacting proteins through plasmid re-transformation eliminates many false positives; a step often dispensed of in high throughput Y2H studies due to the encumbrance of bacterial transformation and plasmid propagation [14]. Yeast plasmids were transformed into *E. coli* DH5α to amplify plasmids. Amplified plasmids were then re-transformed into the yeast strain AH109 (Clonetech) to confirm interactions. Transformed yeast for the secondary screens were first plated on selective medium lacking Leu and Trp. Once yeast colonies appeared, they were then streaked on selective medium lacking His, Leu, and Trp, plus 2 mM 3-AT and medium lacking Ade, Leu, and Trp. Prey plasmids were isolated and sequenced only after confirmation in secondary screens. The PPI datasets were submitted directly to DIP and assigned the International Molecular Exchange identifier IM-15311[55].

### Mating based-split ubiqutin system (mb-SUS) assays

For mating based-split ubitquitin assays, we followed protocols and used vectors and yeast strains as described previously [12]. In brief, using Gateway LR Clonase (Invitrogen) we constructed the bait by transferring XA21cDNA from pENT/D into pMetYC_Gate and the preys through transfer of the corresponding cDNA from pENT/D into pNX_Gate32-3HA. Primers for these constructs are described in Table S10. For identification of positive interaction via yeast mating, the bait and prey constructs were transformed to yeast strain THY.AP5 and THY.AP5, respectively by using the yeast transformation kit, Frozen-EZ yeast transformation II (Zymo Research). Positive interactions were selected by colony growth in minimal SD/Ade-/Leu-/Trp-/His- media (Figure S1).

### Bimolecular fluorescence complementation (BiFC) assays

We conducted BiFC assays as described in Ding et al. [14]. As negative controls, we included the both empty vectors (735 (YC)-EV and 736 (YN)-EV) for each pair-wise test. The BiFC assays are summarized in Table S3 and Figure S2.

### Construction of the co-expression network

We calculated Pearson correlation coefficient (PCC) scores to measure tendency of coexpression between genes based on two sets of publicly available Affymetrix microarray data—219 rice abiotic and 179 rice biotic category data—for 37,993 genes which have Affymetrix probe set matched, of which 34,016 have unique Affymetrix probe set available and only these genes were included in this database (Table S5). The raw Affymetrix data was downloaded from NCBI Gene Expression Omnibus [56] and EBI ArrayExpress [57]. We processed raw Affymetrix data using the MAS 5.0 R-package. The trimmed mean target intensity of each array was arbitrarily set to 500, and the data were then log_2_ transformed. The Rice Multiple-platform Microarray Element Search was used to map the Affymetrix probesets to rice genes [58]. Distributions of PCC scores of 578,527,120 pairs of rice genes with processed microarrays or with randomized microarrays (by random shuffling of arrays) are summarized in Figure 2A and 2C and Table S5.

### Transcriptional profiling of *Xa2*1-, *Nh1*-, and *Nrr*-overexpressing rice

We grew TaiPei309 (TP309), *Xa21::Xa21* 106-17-3-37, LiaoGeng (LG), *Ubi::Nh1* LG 11, and *Ubi::Nrr* 64 LG plants for six weeks in the greenhouse. We then transferred the plants to a growth chamber set for a 14-h daytime period, a 28/26°C temperature cycle and 90% humidity. We employed the scissors dip method with multiple cuts to inoculate the plants using a suspension (OD_600_ of 0.5) of PXO99 *Xoo*. One and two days after inoculation, mock-inoculated and inoculated leaves were harvested for gene expression profiling using the NSF45K array. The replicate mRNAs for the comparisons of *Ubi::Xa21* TP309 *vs* TP309, *Ubi::Nh1* LG *vs.* LG, and *Ubi::Nrr* LG *vs.* LG were labeled with either Cy3 or Cy5 dyes, resulting in one technical replicate and three biological replicates per genotype pair. Gene expression data were processed as previously described [58]. The microarray data have been deposited to NCBI GEO and have the accession number GSE22112.

## Supporting Information

Figure S1 Validation of physical interactions among interactome members via a mating-based split Ubiquitin system (mbSUS). We tested pair-wise interactions between the full-length XA21 and each of the XB proteins using mbSUS. Met YC and NX32 represents pMetYC_Gate [12] and pNX_Gate32-3HA vector [12], respectively. Each construct was tested with MetYC-empty vector (EV) or NX32-EV controls.
(PDF)

Figure S2 Validation of physical interactions among interactome membersvia bimolecular fluorescence complementation (BiFC). We performed BiFC experiments to validate protein–protein interactions of 29 positive Y2H pairs of the rice stress-response interactome (summarized in Table S3). Shown are positive interactions (from 1 to 18) and a representative negative control (735-YC-K668 + 736-YN-empty). Images were taken 1-2 days after transformation. 735-YC[14] and 736-YN[14] indicate the gateway-converted vectors derived from pSY735 (YFP_C-term_) [11] and pSY736 [11] (YFP_N-term_) vector, respectively.
(PDF)

Figure S3 Differentially expressed interactome components based on specific biotic stress-response (XA21/NH1/NRR) and abiotic stress-response (SUB1A) 45K NSF arrays 1 day after application of stress. The interactome components that show differential expression in a given stress array are shown as filled nodes. Array experiments are described in Figure 2E. Red-filled nodes represent proteins for which transcripts accumulate in *Xoo-* resistant responses, including an *Xa21*-dependent (*Xa21*-TP309 vs TP309) up-regulated gene (*Xa21*), *Nh1*-dependent (Nh1 overexpression vs. LG) up-regulated genes (*Pbz1, Sub1C*, and *Xb3*), *Nrr* -dependent (Nrr overexpression vs. LG) down-regulated genes (*Gip13, Nh1, OsWrky62*, and *Xb11*), *Nh1*-dependent up- and *Nrr*-dependent down-regulated genes (*OsWrky76* and *Nrrh1*), and *Nh1*-dependent up- and *Xa21*-dependent up-regulated gene (*Nrrh2*). The blue-filled-node represents the protein for which transcript amounts diminish in *Xoo-*resistant responses, a *Xa21*-dependent down-regulated gene (*Os01g14810)*. Yellow-filled nodes represent proteins for which transcripts accumulate in *Sub1A*-containing rice (*Sub1A* vs. M202) upon submergence (*OsMpk5*, *OsWrky71*, *Sab9*, and *Xb15*). Green-filled nodes represent proteins for which transcript levels diminish in *Sub1A*-containing rice upon submergence (*Sab16*, *Sab21,* and *Scb2*). In addition, two interactome components showed differential expression patterns in both biotic and abiotic stress-response arrays. *Sab8* (dark blue-filled node) showed *Xa21*- and *Sub1a*-dependent decreased gene expression; whereas, *Grnl1* (purple-filled node) showed *Xa21*- and *Sub1a*-dependent increased gene expression. Nodes depicted as rounded rectangles and diamonds represent kinases and transcription factors, respectively.
(PDF)

Figure S4 *OsMpk5* RNAi Nipponbare displays increased resistance to *Xoo*. (A) Water-soaked disease lesions 14 days post inoculation (dpi) of *OsMpk5* RNAi Nipponbare leaves (plant10) compared to Nipponbare leaves (plant 3). (B) Leaf lesion lengths of *OsMpk5* RNAi Nipponbare lines (numbered) versus Nipponbare (WT-1 through -4) 14 d after *Xoo* inoculation. (-) indicates that the line lacks the transgene and (+) that the line possesses the transgene. (C) Expression of *OsMpk5* mRNA in a null segregant and -*OsMpk5* RNAi Nipponbare line. Primers for genotyping and RT-PCR are listed in Table S10.
(PDF)

Figure S5 *OsMpk12* knockout (ko) Dongjin displays increased susceptibility to *Xoo*. (A) Genome structure of *OsMpk12* with T-DNA insertion sites and genotyping primer positions. F: frward primer, R: reverse primer. T: T-DNA specific reverse primer. Boxes and solid lines indicate exons and introns, respectively. Primers for genotyping and RT-PCR are listed in Table S10. (B) Genotyping results for *osmpk12* ko lines (C) Expression of *OsMpk12* mRNA in Dongjin and Donjin-*osmpk12* ko lines. (D) Water-soaked disease lesions 14 days post inoculation (dpi) of *osmpk12* ko Dongjin leaves (plant 1 and 2) compared to Dongjin leaves. (E) *Xoo* population growth over 8 days of infection from three representative leaves per time point from *osmpk12* ko Dongjin vs. Dongjin.
(PDF)

Figure S6 Progeny of *rar1* knockout (ko) Dongjin x *Xa21* monogenic IRBB21 display increased susceptibility to *Xoo*. (A) Genome structure of *RAR1* with T-DNA insertion sites. F; position of forward primer, R; position of reverse primer. T; T-DNA specific reverse primer. Boxes and solid lines indicate exon and intron, respectively. (B) Expression of *RAR1* mRNA in Donjin, *Xa21* (IRBB21), and *rar1* ko X *Xa21* (IRBB21) lines. (C) Genotyping results of F_3_ progeny of *rar1* ko Donjin X *Xa21* (IRBB21) cross. (D) Lesion length results of segregating F_3_ plants. Primers for genotyping are listed in Table S10.
(PDF)

Figure S7 Progeny of *Sab23* overexpression (ox) Dongjin x *Xa21* monogenic IRBB21 display increased susceptibility to *Xoo*. (A) Genotyping results of *Ubi::Sab23* Dongjin X *Xa21* (IRBB21) F_3_ segregants. (B) Lesion length results of segregating F_3_ plants 16 d after *Xoo* inoculation. (C) Expression of *Sab23* mRNA in Donjin, *Xa21* (IRBB21), and *Ubi::Sab23* Dongjin X *Xa21* (IRBB21) F_3_ segregants. Primers for genotyping and RT-PCR are listed in Table S10.
(PDF)

Figure S8 *Scb3* overexpression (ox) Liao Geng (LG) displays increased resistance to *Xoo*. (A) Water-soaked disease lesions 14 days post inoculation (dpi) of *Ubi*::*Scb3* LG leaves (plant 2-1) compared to LG leaves. Water-soaked disease regions on leaves from two genotypes (LG and *Scb3* ox LG line 23-2) 14 d after *Xoo* inoculation (B) Leaf lesion lengths of T_1_ progeny of *Scb3* ox LG lines 14 d after *Xoo* inoculation. (-) indicates that the line lacks the transgene and (+) that the line possesses the transgene. (C) Expression of *Scb3* mRNA in LG and *Scb3* ox LG lines. Primers for genotyping and RT-PCR are listed in Table S11.
(PDF)

Figure S9 *SnRk1a* RNAi, *Xa21*-Kitaake (Kit) displays increased susceptibility to *Xoo*. (A) Water-soaked disease lesions 14 days post inoculation (dpi) of *SnRk1a* RNAi/*Xa21*- Kit leaves (plant 10) compared to Kit and *Xa21*-Kit leaves (plant 3). (B) Leaf lesion lengths of T_1_ progenies of *SnRk1a* RNAi/*Xa21*-Kit lines 14 d after *Xoo* inoculation. (-) indicates that the line lacks the transgene and (+) that the line possesses the transgene. (C) Expression of *SnRk1a* mRNA in *Xa21*-Kit and *SnRk1a* RNAi/*Xa21*-Kit lines. Primers for RT-PCR are listed in Table S10.
(PDF)

Figure S10 *Wak25* overexpression (ox), *Xa21-*Kitaake (Kit) and *Wak25* RNAi, *Xa21*-Kit display increased resistance and increased susceptibility to *Xoo*, respectively. (A) Water-soaked disease lesions 14 dpi of *Ubi*::*Wak25*/*Xa21-*Kit leaves (plant 5-4) and *WaK25* RNAi/*Xa21*-Kit leaves (plant 5-1) compared to *Xa21-*Kit *and* Kit leaves. (B) Expression of *WaK25* mRNA in *Xa21*-Kit, *WaK25* RNAi/*Xa21*-Kit, and *Ubi*::*Wak25*/*Xa21* lines. (C) Leaf lesion lengths of T_1_ progenies of *Ubi*::*Wak25*/*Xa21* lines 14 d after *Xoo* inoculation. (-) indicates that the line lacks the transgene and (+) that the line possesses the transgene. (D) Leaf lesion lengths of T_1_ progeny of *WaK25* RNAi/*Xa21*-Kit (line 5) 14 d after *Xoo* inoculation. (-) indicates that the line lacks the transgene and (+) that the line possesses the transgene. Primers for genotyping and RT-PCR are listed in Table S10.
(PDF)

Figure S11 *OsWrky76* overexpression (ox), *Xa21-*Kitaake (Kit) displays increased susceptibility to *Xoo*. (A) Leaf lesion lengths of T_1_ progeny of *Ubi*::*Wrky76*/*Xa21* Kit plants 14 d after *Xoo* inoculation. (B) Expression of *OsWrky76* mRNA in *Ubi*::*Wrky76*/*Xa21*-Kit lines and *Xa21*-Kit. (-) indicates that the line lacks the transgene and (+) that the line possesses the transgene. Primers for genotyping and RT-PCR are listed in Table S10.
(PDF)

Figure S12 *OsErebp1* overexpression (ox) Kitaake (Kit) displays increased resistance to *Xoo*. (A) Water-soaked disease lesions 14 dpi of T_2_ progenies *Ubi*::*OsErebp1* Kit leaves (plant 4-3-1 and 2-4-1) compared to Kit leaves (B) *Xoo* population growth over 14 days of infection from *Ubi*::*OsErebp1* Kit vs. Kit. (C) Expression of *OsErebp1* mRNA in Kit and *Ubi*::*OsErebp1* Kit. Primers for genotyping and RT-PCR are listed in Table S10.
(PDF)

Figure S13 *sab18* knockout (ko) Dongjin displays decreased elongation in response to submergence. (A) Genome structure of *Sab18* with T-DNA insertion sites. Boxes and solid lines indicate exon and intron, respectively. (B) Genotyping results of *sab18* Dongjin 9-4 line. We also identified another homozygous ko line 9-5, three hetero ko lines (9-7, 9-8, and 9-9) and two null segregants (9-2 and 9-6) (data not shown). (C) Expression of *Sab18* mRNA in *Donjin* and *sab18* Dongjin homozygous ko line 9-4. (D) Plant heights of sab18 Dongjin homozygous ko line 9-4 and *Donjin* 14 d after submergence. Primers for genotyping and RT-PCR are listed in Table S10.
(PDF)

Table S1 100 components of rice stress resposne interactome.
(XLSX)

Table S2 Enrichment of Biological Process Gene Ontology (GO) among 100 network members.
(XLSX)

Table S3 Summary of BiFC experiments*.
(XLSX)

Table S4 Matrix-based protein protein interaction results.
(XLSX)

Table S5 Co-expression analysis from selected public Affymetrix array.
(XLSX)

Table S6 Enrichment in of rice stress-response interactome members in NSF45K array datasets for Xoo or submergence stress.
(XLSX)

Table S7 Summary of phenotypes measured This Study and literature-derived data in XA21/NH1/SUB1 interactome.
(XLSX)

Table S8 Degee in Different Abiotic Gene Exprssion Networks of Interactome Transcripts.
(XLSX)

Table S9 Analysis of GO biological process from XA21, NH1, or NRR interactome via NSF 45K array.
(XLSX)

Table S10 Sequences of forward (F) and reverse (R) primers used in this work.
(XLSX)

Text S1 Y2H plasmid construction and experimental matrix, Construction of binary vectors and generation of transgenic plants, and phenotypic evaluation of transgenic lines with modified expression of interactome members.
(DOCX)
