# Supplementary material for: Towards Establishment of a Rice Stress Response Interactome
Source: PLoS Genet. 2011 Apr 14;7(4):e1002020. doi: 10.1371/journal.pgen.1002020 (PMC3077385; doi:10.1371/journal.pgen.1002020)
Supplement: Figure S1 — Validation of physical interactions among interactome members via a mating-based split Ubiquitin system (mbSUS). We tested pair-wise interactions between the full-length XA21 and each of the XB proteins using mbSUS. Met YC and NX32 represents pMetYC_Gate [12] and pNX_Gate32-3HA vector [12], respectively. Each construct was tested with MetYC-empty vector (EV) or NX32-EV controls. (PDF) [file pgen.1002020.s001.pdf]

Figure S1

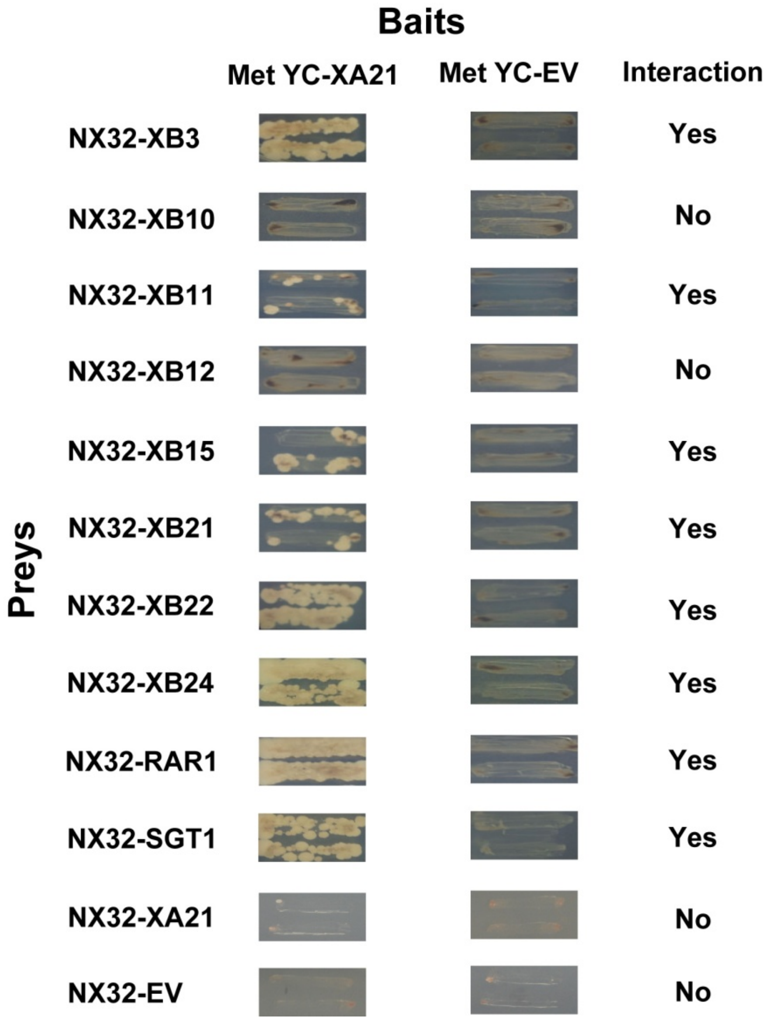
Baits
Met YC-XA21
Met YC-EV
Interaction
Preys
NX32-XB3
Yes
NX32-XB10
No
NX32-XB11
Yes
NX32-XB12
No
NX32-XB15
Yes
NX32-XB21
Yes
NX32-XB22
Yes
NX32-XB24
Yes
NX32-RAR1
Yes
NX32-SGT1
Yes
NX32-XA21
No
NX32-EV
No
