# Supplementary figures and images for: Towards Establishment of a Rice Stress Response Interactome
Source: PLoS Genet. 2011 Apr 14;7(4):e1002020. doi: 10.1371/journal.pgen.1002020 (PMC3077385; doi:10.1371/journal.pgen.1002020)

# Figure S2

## A Positive interactions with controls

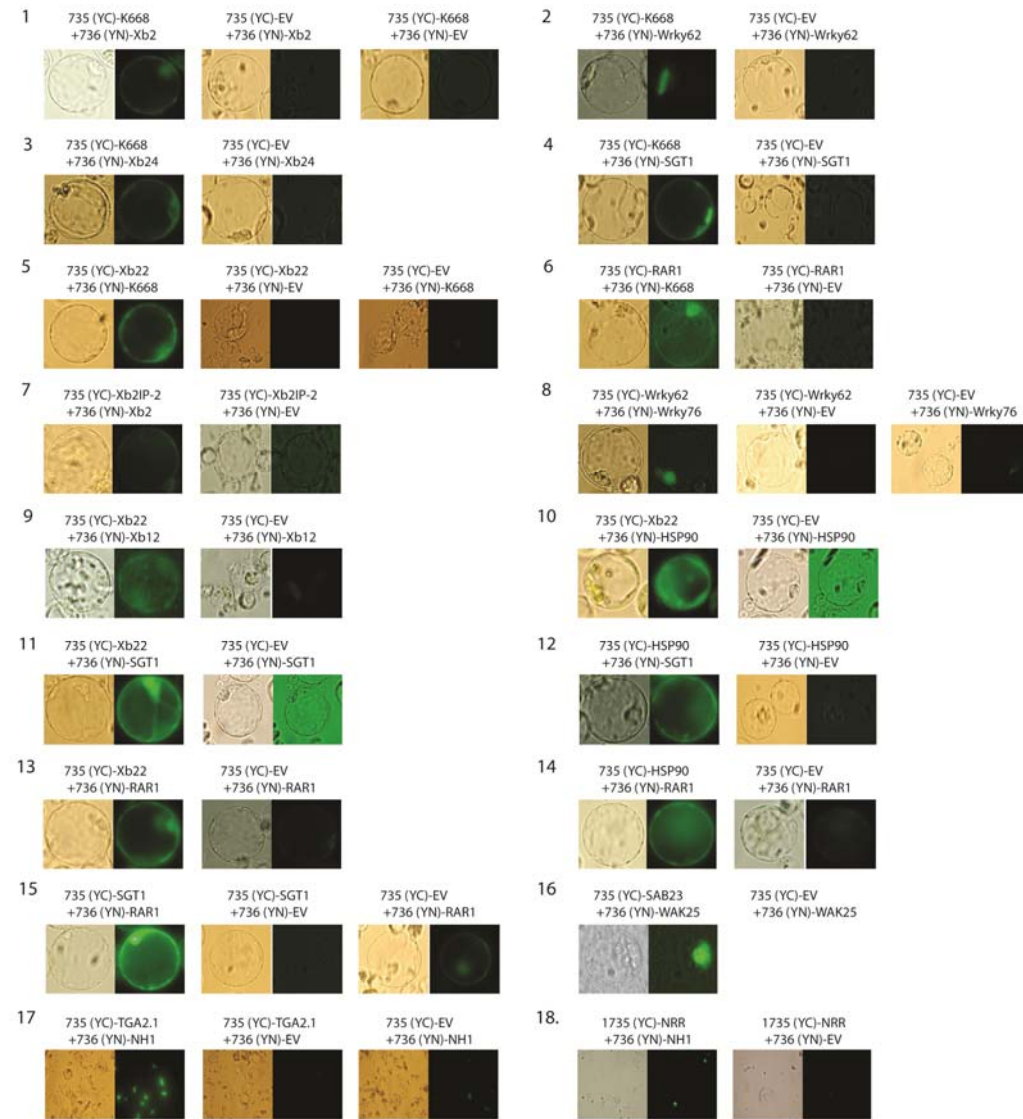

## B Representative negative interactions

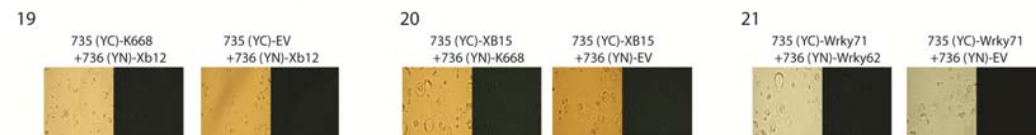

Supplement: Figure S2 — Validation of physical interactions among interactome membersvia bimolecular fluorescence complementation (BiFC). We performed BiFC experiments to validate protein–protein interactions of 29 positive Y2H pairs of the rice stress-response interactome (summarized in Table S3). Shown are positive interactions (from 1 to 18) and a representative negative control (735-YC-K668 + 736-YN-empty). Images were taken 1-2 days after transformation. 735-YC[14] and 736-YN[14] indicate the gateway-converted vectors derived from pSY735 (YFPC-term) [11] and pSY736 [11] (YFPN-term) vector, respectively. (PDF) [file pgen.1002020.s002.pdf]

Figure S4

A

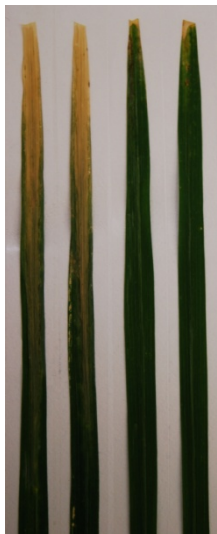

|               |             |           |
|---------------|-------------|-----------|
|               | <u>WT-3</u> | <u>10</u> |
| <i>Osmpk5</i> | +           | RNAi      |
| <i>Osmpk5</i> | +           | RNAi      |

B

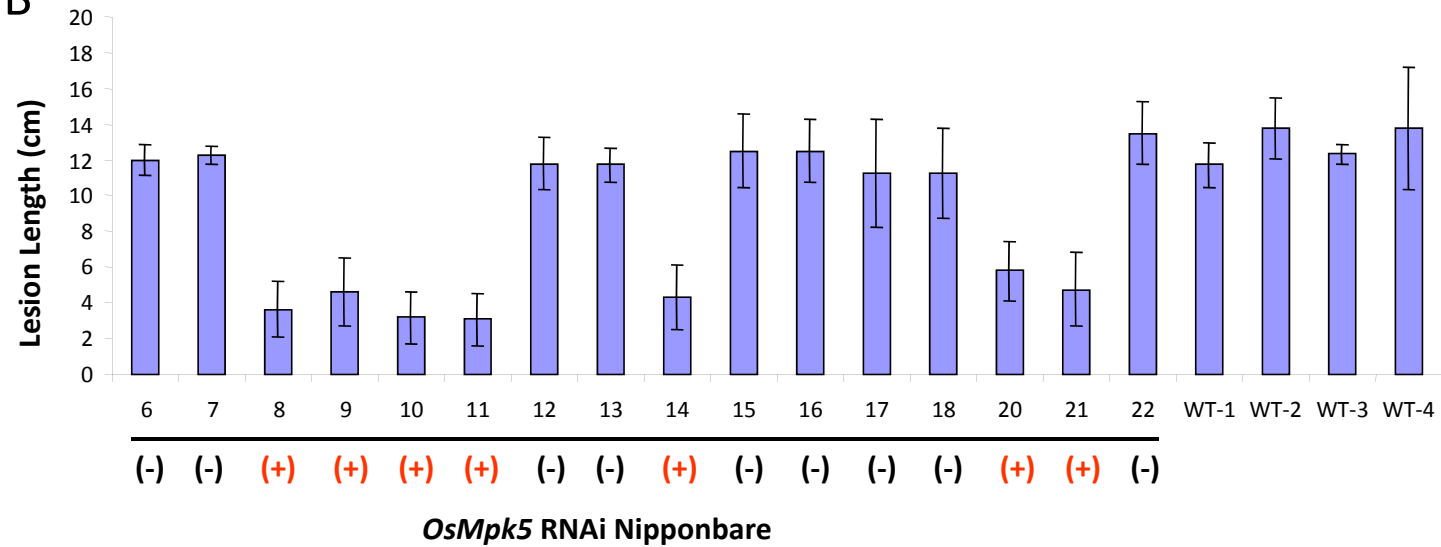

C

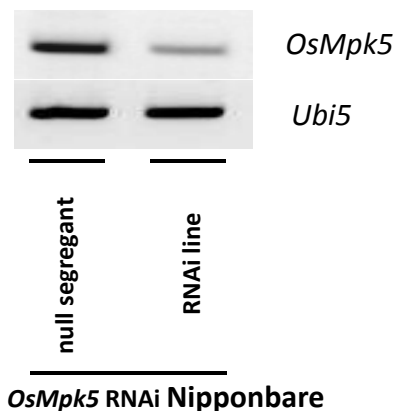

Supplement: Figure S4 — OsMpk5 RNAi Nipponbare displays increased resistance to Xoo. (A) Water-soaked disease lesions 14 days post inoculation (dpi) of OsMpk5 RNAi Nipponbare leaves (plant10) compared to Nipponbare leaves (plant 3). (B) Leaf lesion lengths of OsMpk5 RNAi Nipponbare lines (numbered) versus Nipponbare (WT-1 through -4) 14 d after Xoo inoculation. (-) indicates that the line lacks the transgene and (+) that the line possesses the transgene. (C) Expression of OsMpk5 mRNA in a null segregant and -OsMpk5 RNAi Nipponbare line. Primers for genotyping and RT-PCR are listed in Table S10. (PDF) [file pgen.1002020.s004.pdf]

Figure S5

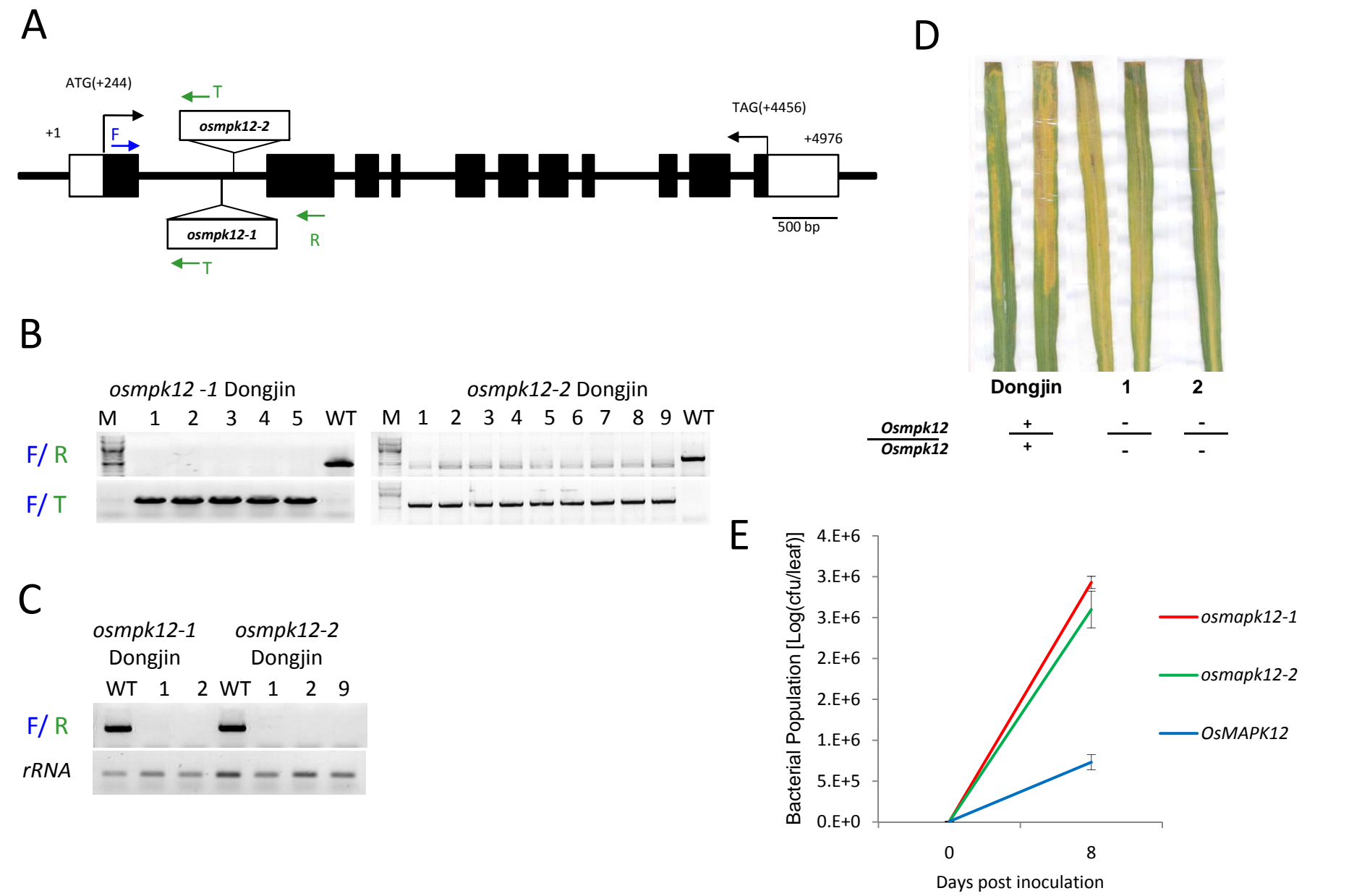

Supplement: Figure S5 — OsMpk12 knockout (ko) Dongjin displays increased susceptibility to Xoo. (A) Genome structure of OsMpk12 with T-DNA insertion sites and genotyping primer positions. F: frward primer, R: reverse primer. T: T-DNA specific reverse primer. Boxes and solid lines indicate exons and introns, respectively. Primers for genotyping and RT-PCR are listed in Table S10. (B) Genotyping results for osmpk12 ko lines (C) Expression of OsMpk12 mRNA in Dongjin and Donjin-osmpk12 ko lines. (D) Water-soaked disease lesions 14 days post inoculation (dpi) of osmpk12 ko Dongjin leaves (plant 1 and 2) compared to Dongjin leaves. (E) Xoo population growth over 8 days of infection from three representative leaves per time point from osmpk12 ko Dongjin vs. Dongjin. (PDF) [file pgen.1002020.s005.pdf]

**Figure S6**

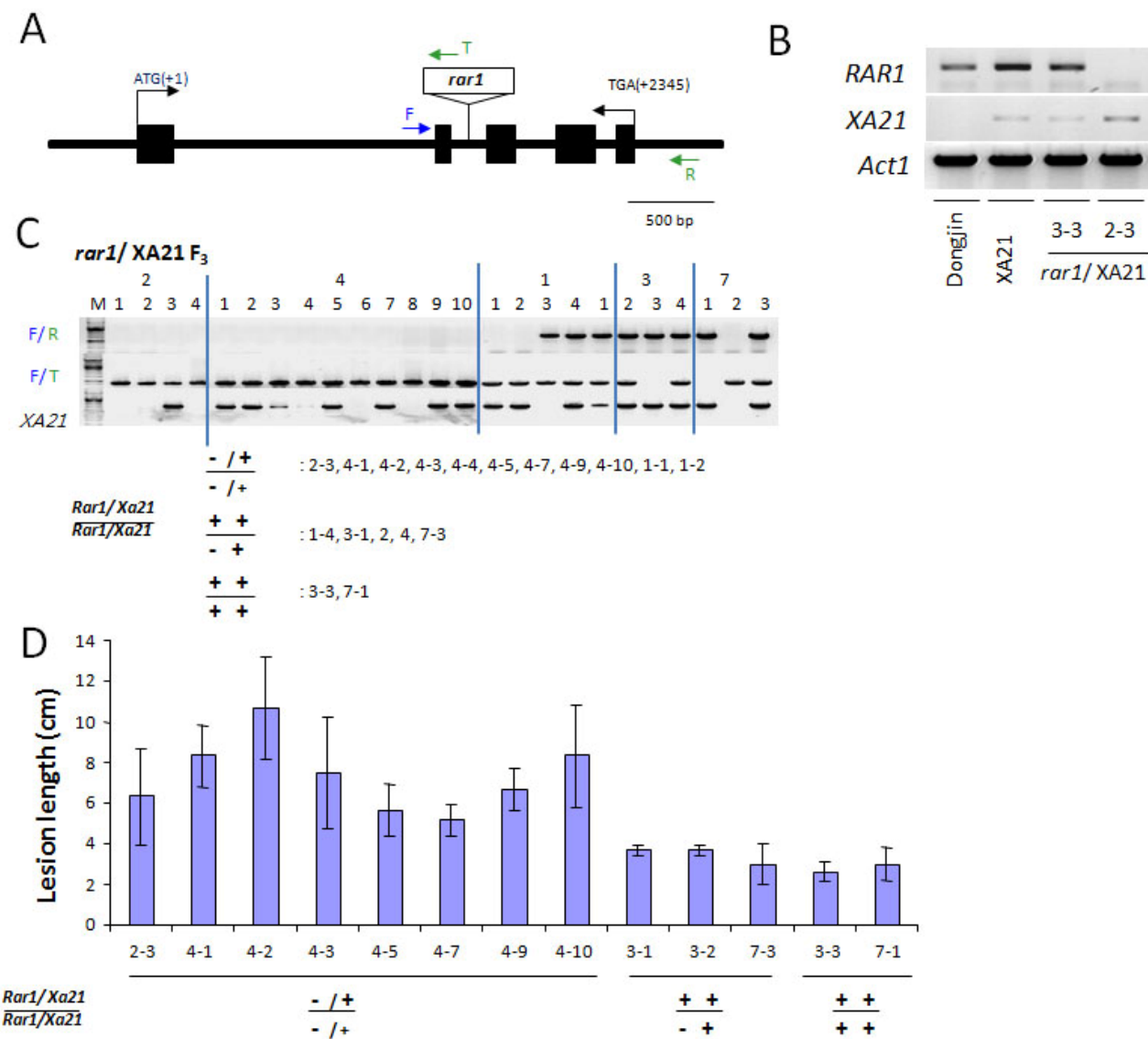

Supplement: Figure S6 — Progeny of rar1 knockout (ko) Dongjin x Xa21 monogenic IRBB21 display increased susceptibility to Xoo. (A) Genome structure of RAR1 with T-DNA insertion sites. F; position of forward primer, R; position of reverse primer. T; T-DNA specific reverse primer. Boxes and solid lines indicate exon and intron, respectively. (B) Expression of RAR1 mRNA in Donjin, Xa21 (IRBB21), and rar1 ko X Xa21 (IRBB21) lines. (C) Genotyping results of F3 progeny of rar1 ko Donjin X Xa21 (IRBB21) cross. (D) Lesion length results of segregating F3 plants. Primers for genotyping are listed in Table S10. (PDF) [file pgen.1002020.s006.pdf]

**Figure S7**

**A**

*Sab23* ox / *Xa21* F<sub>3</sub>

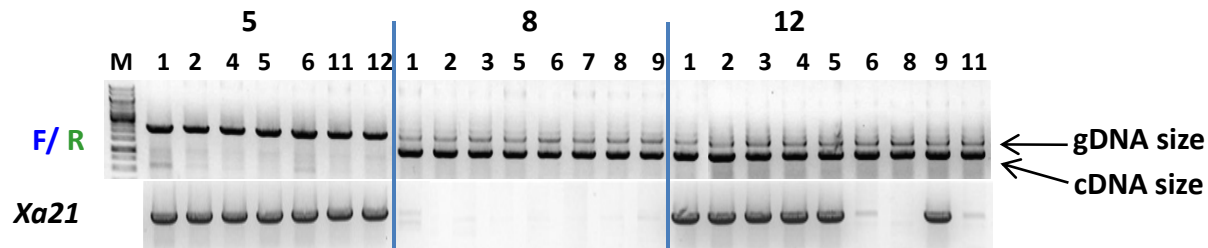

**B**

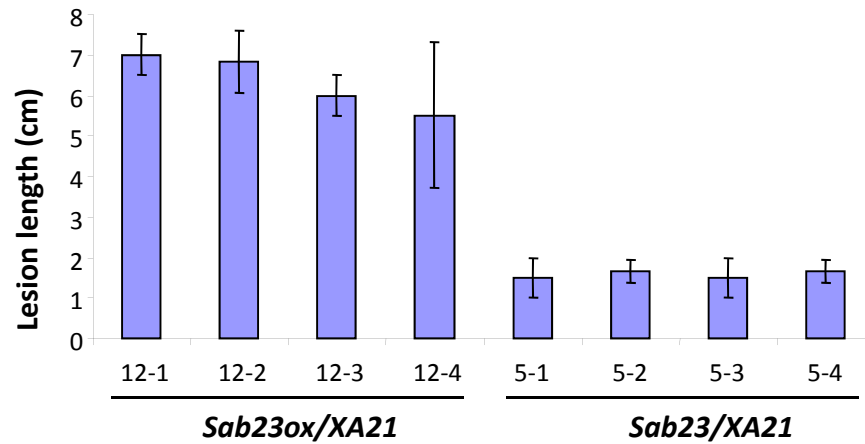

**C**

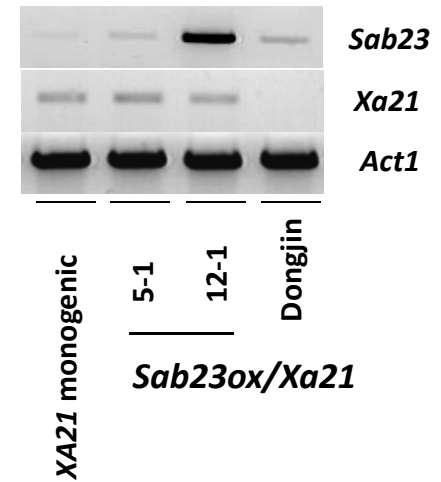

Supplement: Figure S7 — Progeny of Sab23 overexpression (ox) Dongjin x Xa21 monogenic IRBB21 display increased susceptibility to Xoo. (A) Genotyping results of Ubi::Sab23 Dongjin X Xa21 (IRBB21) F3 segregants. (B) Lesion length results of segregating F3 plants 16 d after Xoo inoculation. (C) Expression of Sab23 mRNA in Donjin, Xa21 (IRBB21), and Ubi::Sab23 Dongjin X Xa21 (IRBB21) F3 segregants. Primers for genotyping and RT-PCR are listed in Table S10. (PDF) [file pgen.1002020.s007.pdf]

Figure S8

A

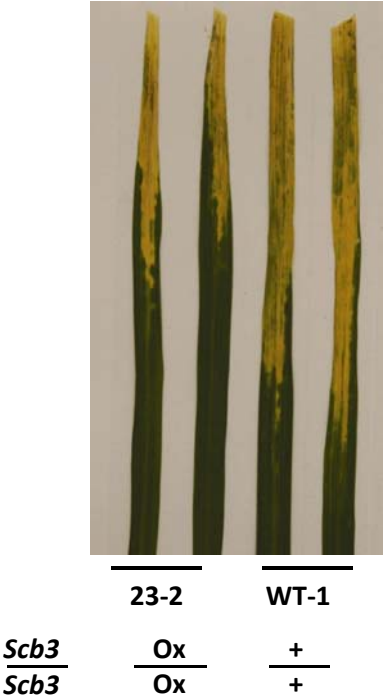

B

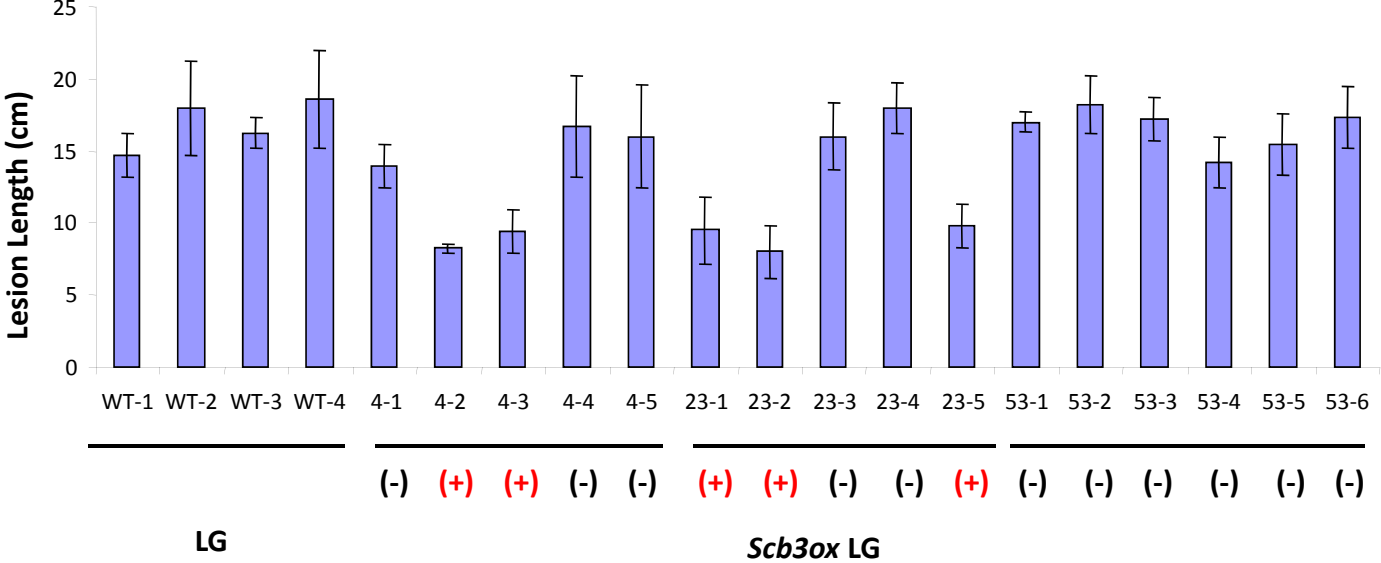

C

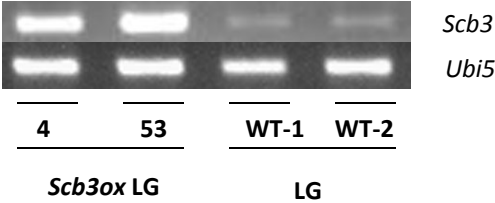

Supplement: Figure S8 — Scb3 overexpression (ox) Liao Geng (LG) displays increased resistance to Xoo. (A) Water-soaked disease lesions 14 days post inoculation (dpi) of Ubi::Scb3 LG leaves (plant 2-1) compared to LG leaves. Water-soaked disease regions on leaves from two genotypes (LG and Scb3 ox LG line 23-2) 14 d after Xoo inoculation (B) Leaf lesion lengths of T1 progeny of Scb3 ox LG lines 14 d after Xoo inoculation. (-) indicates that the line lacks the transgene and (+) that the line possesses the transgene. (C) Expression of Scb3 mRNA in LG and Scb3 ox LG lines. Primers for genotyping and RT-PCR are listed in Table S11. (PDF) [file pgen.1002020.s008.pdf]

Figure S9

A

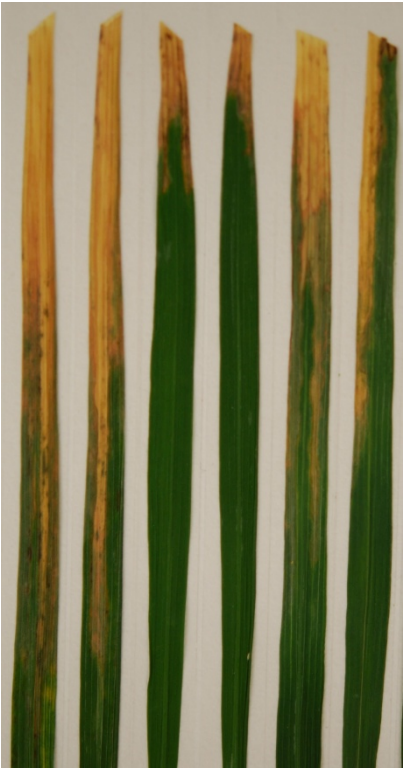

Kitakke      Kit-XA21      2-6

|                   |             |   |   |   |   |      |   |
|-------------------|-------------|---|---|---|---|------|---|
| <i>SnRk1a</i>     | <i>Xa21</i> | + | - | + | + | RNAi | + |
| <i>SnRk1aXa21</i> |             | + | - | + | + | RNAi | + |

B

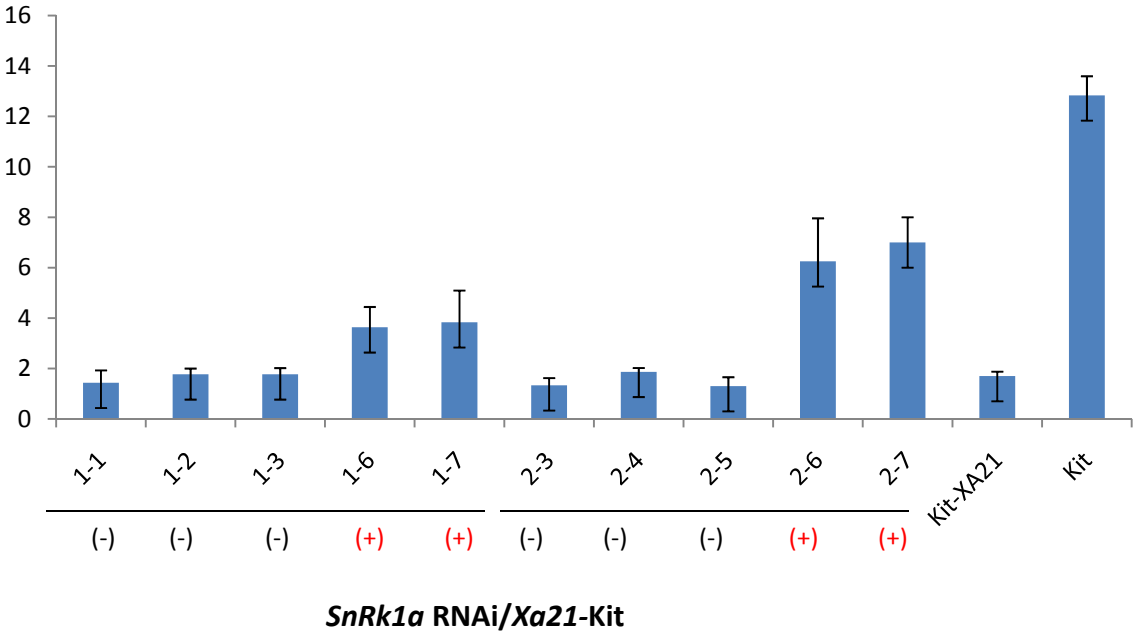

C

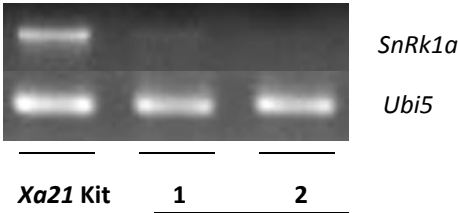

*SnRk1a* RNAi/*Xa21*-Kit

Supplement: Figure S9 — SnRk1a RNAi, Xa21-Kitaake (Kit) displays increased susceptibility to Xoo. (A) Water-soaked disease lesions 14 days post inoculation (dpi) of SnRk1a RNAi/Xa21- Kit leaves (plant 10) compared to Kit and Xa21-Kit leaves (plant 3). (B) Leaf lesion lengths of T1 progenies of SnRk1a RNAi/Xa21-Kit lines 14 d after Xoo inoculation. (-) indicates that the line lacks the transgene and (+) that the line possesses the transgene. (C) Expression of SnRk1a mRNA in Xa21-Kit and SnRk1a RNAi/Xa21-Kit lines. Primers for RT-PCR are listed in Table S10. (PDF) [file pgen.1002020.s009.pdf]

Figure S10

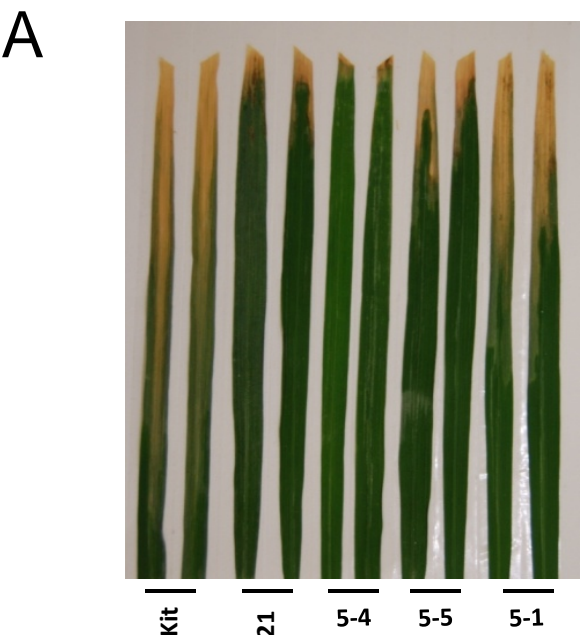

|              |             |   |   |   |   |    |   |   |   |   |      |   |
|--------------|-------------|---|---|---|---|----|---|---|---|---|------|---|
| <i>Wak25</i> | <i>Xa21</i> | + | - | + | + | Ox | + | + | + | + | RNAi | + |
| <i>Wak25</i> | <i>Xa21</i> | + | - | + | + | Ox | + | + | + | + | RNAi | + |

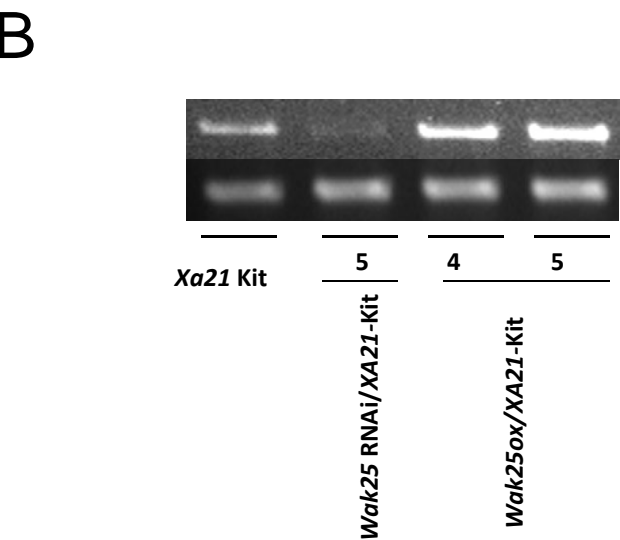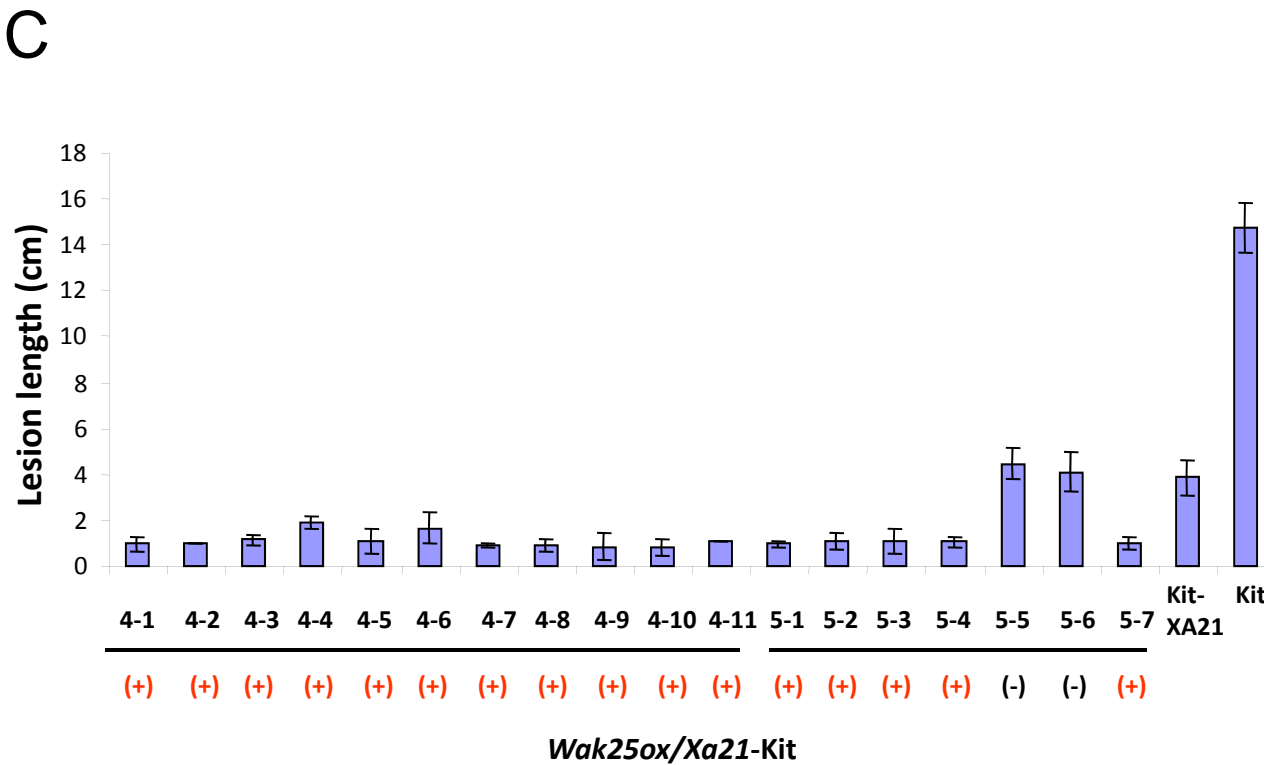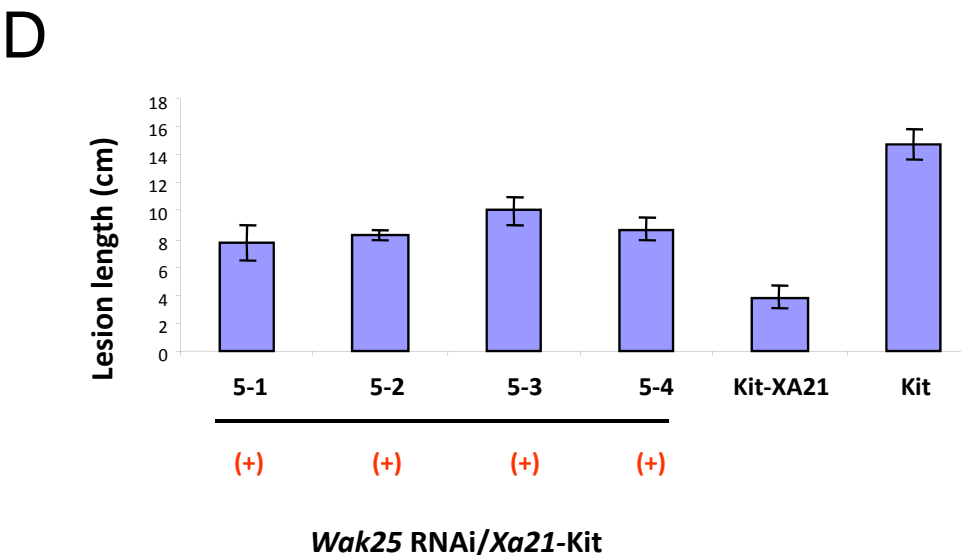

Supplement: Figure S10 — Wak25 overexpression (ox), Xa21-Kitaake (Kit) and Wak25 RNAi, Xa21-Kit display increased resistance and increased susceptibility to Xoo, respectively. (A) Water-soaked disease lesions 14 dpi of Ubi::Wak25/Xa21-Kit leaves (plant 5-4) and WaK25 RNAi/Xa21-Kit leaves (plant 5-1) compared to Xa21-Kit and Kit leaves. (B) Expression of WaK25 mRNA in Xa21-Kit, WaK25 RNAi/Xa21-Kit, and Ubi::Wak25/Xa21 lines. (C) Leaf lesion lengths of T1 progenies of Ubi::Wak25/Xa21 lines 14 d after Xoo inoculation. (-) indicates that the line lacks the transgene and (+) that the line possesses the transgene. (D) Leaf lesion lengths of T1 progeny of WaK25 RNAi/Xa21-Kit (line 5) 14 d after Xoo inoculation. (-) indicates that the line lacks the transgene and (+) that the line possesses the transgene. Primers for genotyping and RT-PCR are listed in Table S10. (PDF) [file pgen.1002020.s010.pdf]

Figure S11

A

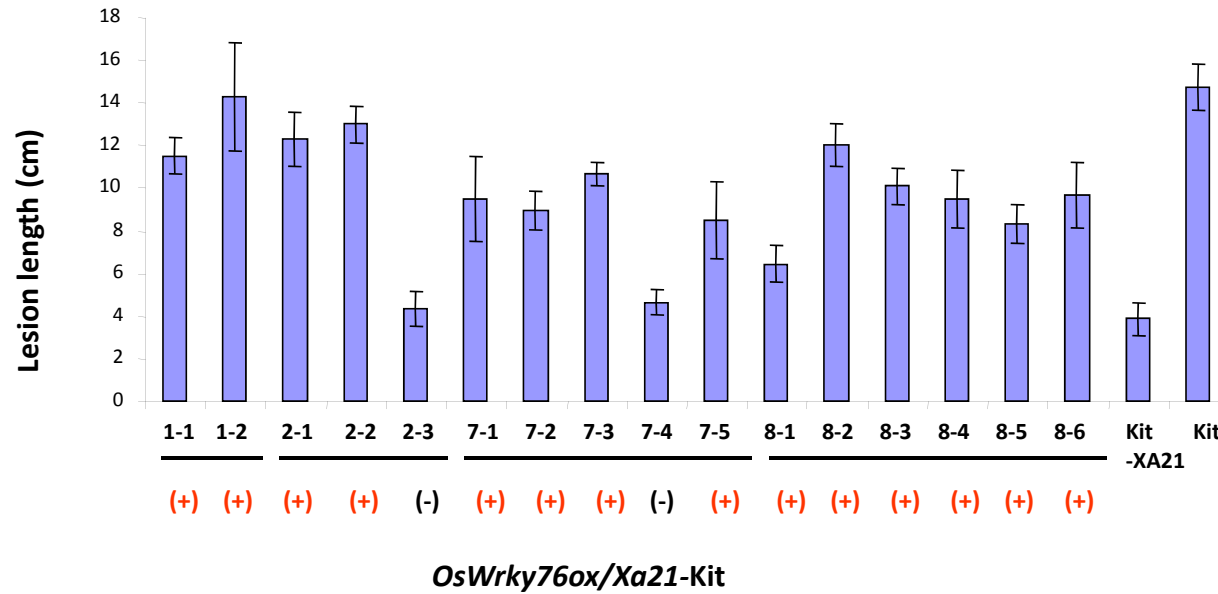

B

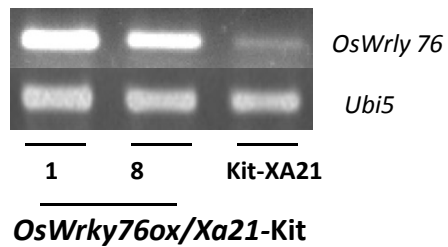

Supplement: Figure S11 — OsWrky76 overexpression (ox), Xa21-Kitaake (Kit) displays increased susceptibility to Xoo. (A) Leaf lesion lengths of T1 progeny of Ubi::Wrky76/Xa21 Kit plants 14 d after Xoo inoculation. (B) Expression of OsWrky76 mRNA in Ubi::Wrky76/Xa21-Kit lines and Xa21-Kit. (-) indicates that the line lacks the transgene and (+) that the line possesses the transgene. Primers for genotyping and RT-PCR are listed in Table S10. (PDF) [file pgen.1002020.s011.pdf]

Figure S12

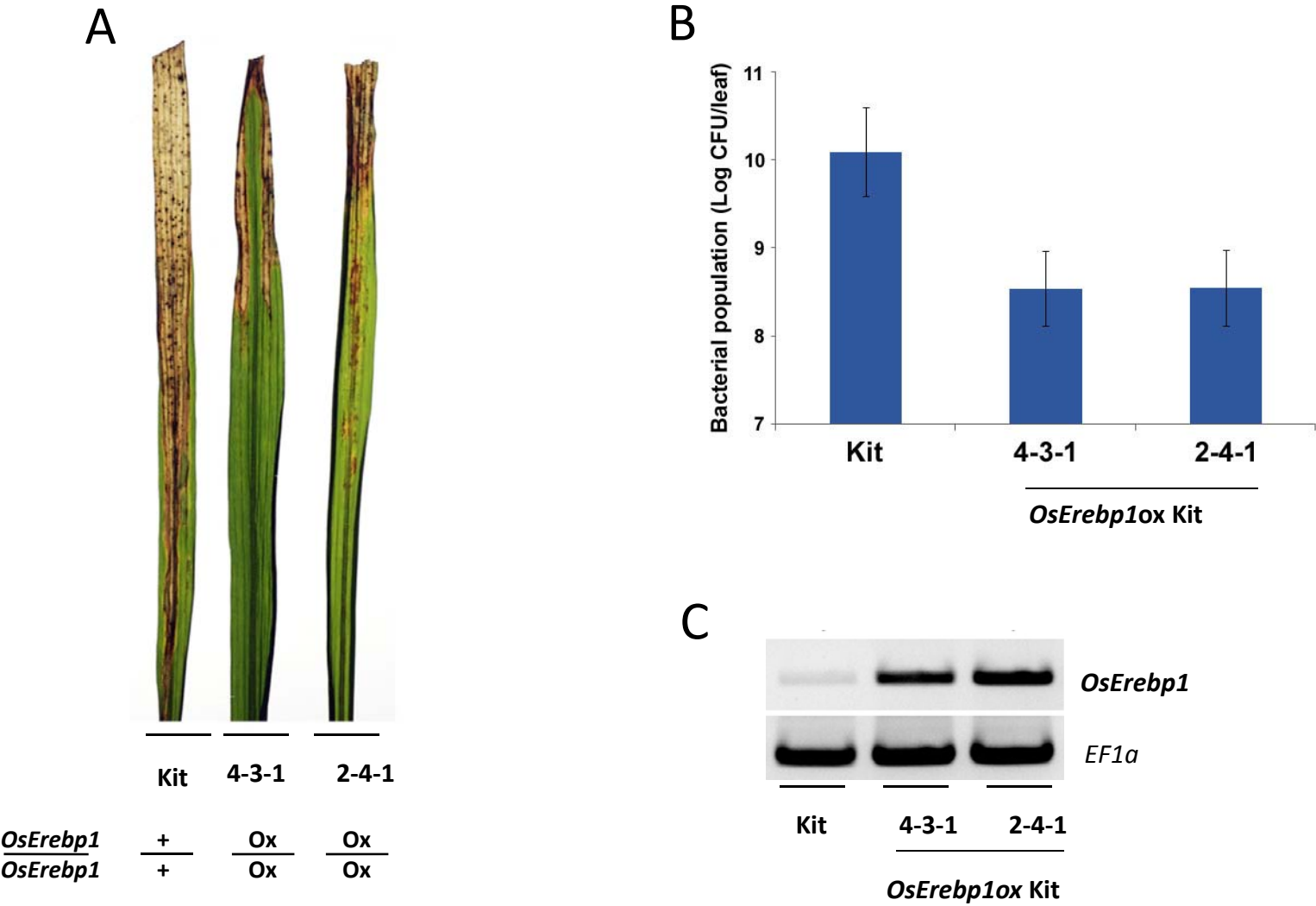

Supplement: Figure S12 — OsErebp1 overexpression (ox) Kitaake (Kit) displays increased resistance to Xoo. (A) Water-soaked disease lesions 14 dpi of T2 progenies Ubi::OsErebp1 Kit leaves (plant 4-3-1 and 2-4-1) compared to Kit leaves (B) Xoo population growth over 14 days of infection from Ubi::OsErebp1 Kit vs. Kit. (C) Expression of OsErebp1 mRNA in Kit and Ubi::OsErebp1 Kit. Primers for genotyping and RT-PCR are listed in Table S10. (PDF) [file pgen.1002020.s012.pdf]

Figure S13

A

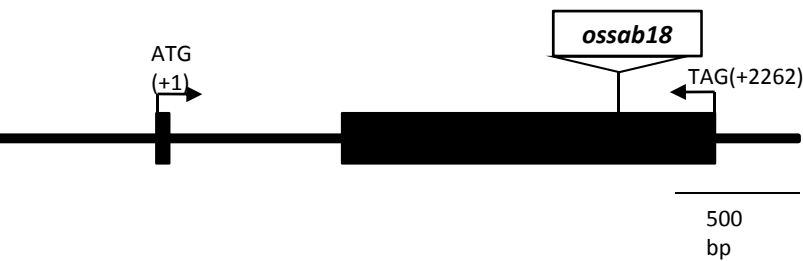

B

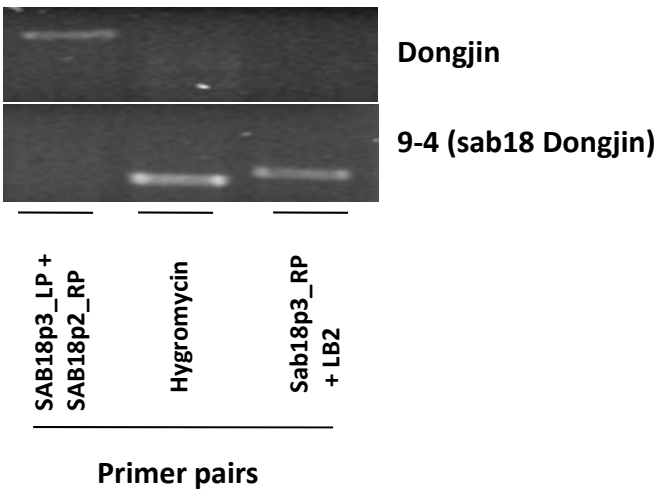

C

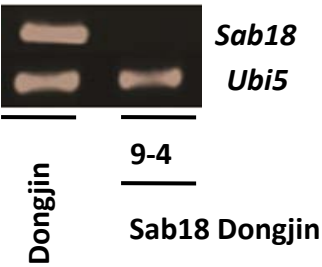

D

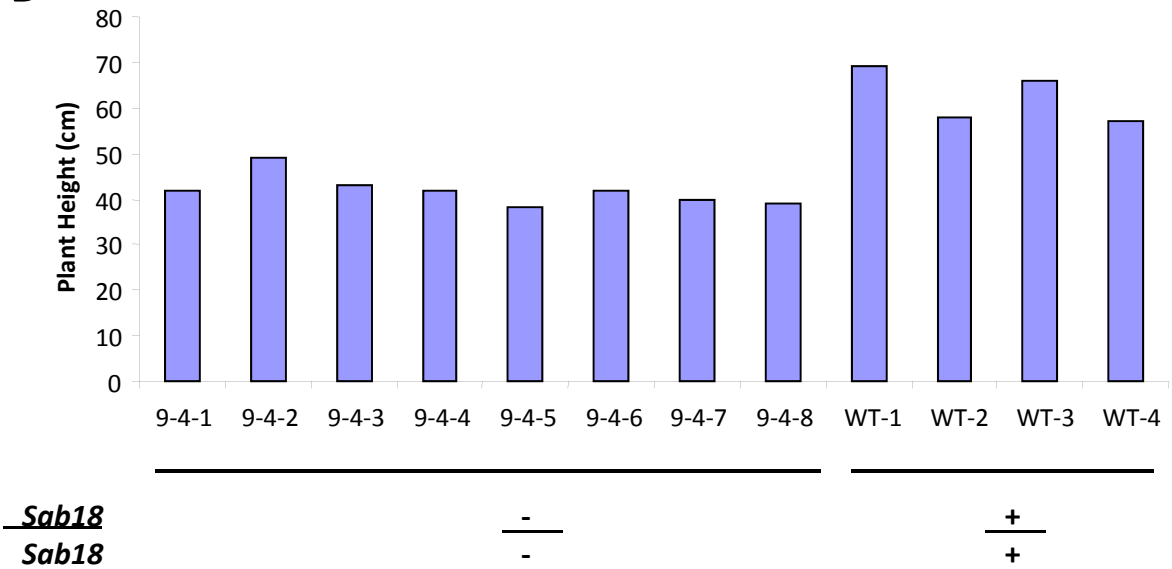

Supplement: Figure S13 — sab18 knockout (ko) Dongjin displays decreased elongation in response to submergence. (A) Genome structure of Sab18 with T-DNA insertion sites. Boxes and solid lines indicate exon and intron, respectively. (B) Genotyping results of sab18 Dongjin 9-4 line. We also identified another homozygous ko line 9-5, three hetero ko lines (9-7, 9-8, and 9-9) and two null segregants (9-2 and 9-6) (data not shown). (C) Expression of Sab18 mRNA in Donjin and sab18 Dongjin homozygous ko line 9-4. (D) Plant heights of sab18 Dongjin homozygous ko line 9-4 and Donjin 14 d after submergence. Primers for genotyping and RT-PCR are listed in Table S10. (PDF) [file pgen.1002020.s013.pdf]
