# Supplementary material for: Towards Establishment of a Rice Stress Response Interactome
Source: PLoS Genet. 2011 Apr 14;7(4):e1002020. doi: 10.1371/journal.pgen.1002020 (PMC3077385; doi:10.1371/journal.pgen.1002020)
Supplement: Text S1 — Y2H plasmid construction and experimental matrix, Construction of binary vectors and generation of transgenic plants, and phenotypic evaluation of transgenic lines with modified expression of interactome members. (DOCX) [file pgen.1002020.s024.docx]

**Text S1**

**Y2H plasmid construction and experimental matrix**

The cDNA for each gene tested was amplified from a rice cDNA pool or a Rice Genome Researh Center cDNA clone [1] (<http://cdna01.dna.affrc.go.jp/cDNA/>, primers described in **Table S10) and was cloned into pENTR/D TOPO (Invitrogen) according to the instructions provided by the manufacturer. The positive clones were verified by sequencing and then, using Gateway LR Clonase (Invitrogen), moved into the Y2H vector pNlexA carrying the BD domain (Clontech) or pB42AD containing AD domain (Clontech). The purified plasmid DNAs from BD vector-bait genes and AD vector-prey genes were transformed into the yeast, pEGY48/p8op-LacZ (Clontech), using the Yeast transformation kit, Frozen-EZ yeast transformation II (Zymo Research). Positive interaction was determined by color development of yeast two days after streaking on media containing X-gal [2].**

**Due to autoactivation of XB15, XB21, XB24, XB12IP-1, XB12IP-4 (hereafter, OsWRKY67) and** OsEREBP1 (**from the XA21 interactome) and SAB1and SAB2 (from the SUB1 interactome), we exclude the use of these pNLexA constructs. Difficulty in construction of some vectors, such as pB42AD-WAK25, pB42AD-XB2IP-1, pB42AD-OsWRKY76, p42AD-XA21K668, pB42AD-SUB1A, pB42AD-SUB1C, pNlexA-OsWRKY76 and pNlexA-XB12IP-1, prevented us from using these constructs. Thus, we tested the pair-wise protein-protein interactions (PPI) in a 20 (pNLexA-constructs) x 24 (p42AD-constructs) matrix of 27 biotic stress response interactome (XA21 interactome) components and a 14 (pNLexA-constructs) x 14 (p42AD-constructs) matrix of 16 abiotic stress response interactome (SUB1 interactome) components. For a matrix of biotic-abiotic stress response interactome components, we tested pair-wise PPI in a 24 (XA21 interactome ) x 16 (SUB1 interactome) matrix based on constructs available. The lists of tested interactions in each matrix are in Table S4-A.**

**Construction of binary vectors and generation of transgenic plants**

With the exception of the *Sab23* overexpression vector, we constructed binary vectors for overexpression by recombining cloned cDNAs **using Gateway LR Clonase (Invitrogen) into the Ubi-NC1300-Rfa vector [3], in which the Gateway cassette is flanked by the maize *ubiquitin 1* promoter including the first intron, and the 3´-terminator of the *nopaline synthase* gene from *Agrobacterium tumefaciens*. Hygromycin is the selectable marker for this vector. To construct the *Sab23* overexpression vector,** the full-length cDNA of *Sab23* was PCR amplified with a pair of primers containing *Xba*I and *Bam*HI site (**Table S10**). The product was cloned into the pGEM®-T Vector (Promega) and sequenced. Then the fragment was excised via digestion and cloned into the *Xba*I and *Bam*HI site of pPZPIIHa3, which contains the cauliflower mosaic virus *35S* promoter and **3´-terminator of the *nopaline synthase* gene from *Agrobacterium tumefaciens*. Hygromycin is the selectable marker for this vector.** To construct vectors for RNAi–mediated gene silencing, 350-450 base pair fragments of the cDNA from the targeted gene was amplified (primers described in **Table S10), cloned into pENTR/D TOPO (Invitrogen) according to the instructions provided by the manufacturer, and the insert confirmed by sequencing. This insert was recombined into the final pANDA vector [4] using Gateway LR Clonase (Invitrogen).**

Rice transformation was conducted as described previously [5]. Three to twelve independently transformed plant lines were isolated for each construct. Overexpression constructs for *OsMpk8*, *OsWrky76*, *Xb11ip-2*, and *Wak25* and RNAi construct of *SnRk1a*, *OsWrky28*, *Xb11ip-2*, and *Wak25* were introduced using a hygromycin selectable marker into a homozygous Kitaake-*Xa21* line[3], which possesses a genomic copy of XA21 with its native promoter linked to a mannose selectable marker. We also generated three to five independent transgenic lines in the Kitaake genetic background, pANDA-*Xb11*, *Ubi*::*Xb11ip-1*, and *Ubi*::*OsErebp1*.

**In addition, we generated three independently transformed *Sab23* overexpression lines in the** Japonica cultivar, Dongjin. To study SAB23 function in XA21-mediated immunity, this line was crossed with an Indica cultivar that possesses the *Xa21* locus, IRBB21. We also generated five independent cross lines between the *rar1* knockout line and IRBB21. For identification of regulators in response to submergence, we used the submergence-sensitive cultivar Liao Geng (LG) to test selected candidate genes, which lacks *Sub1a*.

We also obtained and characterized seven mutant lines from the POSTech T-DNA, indexed rice mutant collection, as described in the Flanking sequence Database [6], which are in the cultivars Dongjin or *Hwayoung*, both of which lack *Xa21* and *Sub1a*. Line 3A-09424 has an insertion in the intron of *OsMpk12*; line 3A-17135 has an insertion in the intron of *Rar1*; line 3A-51026 has an insertion in the 3’-UTR of *OsWrky76*; line 2D-41499 has an insertion in the 5’-UTR of *OsWrky28*; line 3A-16733 has an insertion in an exon of *Sab9*; line 4A-00909 has an insertion in an exon of *Sab16*; line 1B-02315 has an insertion in an exon of *Sab18*. These mutants were confirmed by PCR (primers in Table S10).

**Phenotypic evaluation of transgenic lines with modified expression of interactome members**

**A. Phenotypic evaluation of lines in response to *Xoo***.

To evaluate the response of various lines to *Xoo* inoculation, we inoculated 4-5 week-old plants with *Xoo* via the scissors-dip method [7]. The length of resulting water-soaked lesions was evaluated 14-21 days after inoculation. We evaluated two to five lines for each construct and **the information on the tested lines is in Table S7.** The experiments conducted and results are summarized in Table S6 and Figures S4-12. **In the *Xa21-*containing Kitaake (Kit) genetic background, we did not observe phenotypic changes in any of the independently transformed lines associated with altered expression due to four transgenic constructs (***Ubi*::*Osmpk8, pANDA-Oswrky28, Ubi*::*Xb11ip-2,* and *pANDA-Xb11ip-2***; whereas, we did observe phenotypic changes for three candidate genes (e.g.,** *Ubi*::*Oswrky76***,** *pANDA-Snrk1a***,** *Ubi*::*Wak25***, and** *pANDA-Wak25***) (Table S7).** In the Kit genetic background, *Ubi*::*Oserebp-1* displayed increased resistance to *Xoo*, whereas the other two lines (pANDA-*Xb11*, *Ubi*::*Xb11ip-*1) did not confer any phenotypes to *Xoo*. In the Dongjin genetic background, osmpk12ko displayed increased susceptibility to *Xoo*, whereas two knockout lines such as *oswrky76* ko and *oswrky28* ko did not change phenotypes in response to *Xoo*. In the Dongjin/IRBB21 genetic background, we observed phenotypic changes in *osmpk12 ko*, *rar1*ko/IRBB21, and *Sab23ox*/IRBB21 lines **(Table S7).**

**B. Phenotypic evaluation of lines in response to submergence treatment.**

We used 14-21 day-old plants for submergence experiments. Submergence damages rice foliage by causing accelerated leaf elongation accompanied by weakness, leaf chlorosis and accelerated senescence [8]. *SUB1A* promotes a strategy of quiescence that limits carbohydrate consumption and leaf elongation while maintaining leaf chlorophyll content, leading to plant survival with up to 14 days of submergence[9,10,11,12]. We monitored and measured shoot elongation 6 and 14 days after submergence and plant survival 14 days after submergence, as described previously [11] and we used wild type for each line as control. For most of the lines, we did not observe any alterations in shoot elongation or submergence tolerance in any of the lines. However, the *sab18* ko lines had a marked decrease in shoot elongation, suggesting a role in the response of SAB18 to submergence.

Literature Cited

1. Kikuchi S, Satoh K, Nagata T, Kawagashira N, Doi K, et al. (2003) Collection, mapping, and annotation of over 28,000 cDNA clones from japonica rice. Science 301: 376-379.

2. Chern M, Fitzgerald HA, Canlas PE, Navarre DA, Ronald PC (2005) Overexpression of a rice NPR1 homolog leads to constitutive activation of defense response and hypersensitivity to light. Mol Plant Microbe Interact 18: 511-520.

3. Peng Y, Bartley LE, Chen X, Dardick C, Chern M, et al. (2008) OsWRKY62 is a negative regulator of basal and Xa21-mediated defense against Xanthomonas oryzae pv. oryzae in rice. Mol Plant 1: 446-458.

4. Miki D, Shimamoto K (2004) Simple RNAi vectors for stable and transient suppression of gene function in rice. Plant Cell Physiol 45: 490-495.

5. Chern MS, Fitzgerald HA, Yadav RC, Canlas PE, Dong X, et al. (2001) Evidence for a disease-resistance pathway in rice similar to the NPR1-mediated signaling pathway in Arabidopsis. Plant J 27: 101-113.

6. Jeong DH, An S, Park S, Kang HG, Park GG, et al. (2006) Generation of a flanking sequence-tag database for activation-tagging lines in japonica rice. Plant J 45: 123-132.

7. Song WY, Wang GL, Chen LL, Kim HS, Pi LY, et al. (1995) A receptor kinase-like protein encoded by the rice disease resistance gene, Xa21. Science 270: 1804-1806.

8. Jackson MB, Ram PC (2003) Physiological and molecular basis of susceptibility and tolerance of rice plants to complete submergence. Ann Bot 91 Spec No: 227-241.

9. Fukao T, Bailey-Serres J (2008) Submergence tolerance conferred by Sub1A is mediated by SLR1 and SLRL1 restriction of gibberellin responses in rice. Proc Natl Acad Sci U S A 105: 16814-16819.

10. Fukao T, Xu K, Ronald PC, Bailey-Serres J (2006) A variable cluster of ethylene response factor-like genes regulates metabolic and developmental acclimation responses to submergence in rice. Plant Cell 18: 2021-2034.

11. Jung KH, Seo YS, Walia H, Cao P, Fukao T, et al. (2010) The submergence tolerance regulator Sub1A mediates stress-responsive expression of AP2/ERF transcription factors. Plant Physiol 152: 1674-1692.

12. Xu K, Xu X, Fukao T, Canlas P, Maghirang-Rodriguez R, et al. (2006) Sub1A is an ethylene-response-factor-like gene that confers submergence tolerance to rice. Nature 442: 705-708.
